# Analysis of a Compartmental Model of Endogenous Immunoglobulin G Metabolism with Application to Multiple Myeloma

**DOI:** 10.3389/fphys.2017.00149

**Published:** 2017-03-17

**Authors:** Felicity Kendrick, Neil D. Evans, Bertrand Arnulf, Hervé Avet-Loiseau, Olivier Decaux, Thomas Dejoie, Guillemette Fouquet, Stéphanie Guidez, Stéphanie Harel, Benjamin Hebraud, Vincent Javaugue, Valentine Richez, Susanna Schraen, Cyrille Touzeau, Philippe Moreau, Xavier Leleu, Stephen Harding, Michael J. Chappell

**Affiliations:** ^1^School of Engineering, University of WarwickCoventry, UK; ^2^Hôpital Saint-LouisParis, France; ^3^Unité de Génomique du Myélome, Institut Universitaire du Cancer de Toulouse OncopoleToulouse, France; ^4^Centre Hospitalier Universitaire de RennesRennes, France; ^5^Centre Hospitalier Universitaire de NantesNantes, France; ^6^Centre Hospitalier Régional Universitaire de LilleLille, France; ^7^Centre Hospitalier Universitaire de PoitiersPoitiers, France; ^8^Centre Hospitalier Universitaire de ToulouseToulouse, France; ^9^Centre Hospitalier Universitaire de NiceNice, France; ^10^Department of Research and Development, The Binding Site Group LimitedBirmingham, UK

**Keywords:** biomedical systems, lumped-parameter systems, identifiability, parameter identification, sensitivity analysis, immunoglobulin G, metabolism, multiple myeloma

## Abstract

Immunoglobulin G (IgG) metabolism has received much attention in the literature for two reasons: (i) IgG homeostasis is regulated by the neonatal Fc receptor (FcRn), by a pH-dependent and saturable recycling process, which presents an interesting biological system; (ii) the IgG-FcRn interaction may be exploitable as a means for extending the plasma half-life of therapeutic monoclonal antibodies, which are primarily IgG-based. A less-studied problem is the importance of endogenous IgG metabolism in IgG multiple myeloma. In multiple myeloma, quantification of serum monoclonal immunoglobulin plays an important role in diagnosis, monitoring and response assessment. In order to investigate the dynamics of IgG in this setting, a mathematical model characterizing the metabolism of endogenous IgG in humans is required. A number of authors have proposed a two-compartment nonlinear model of IgG metabolism in which saturable recycling is described using Michaelis–Menten kinetics; however it may be difficult to estimate the model parameters from the limited experimental data that are available. The purpose of this study is to analyse the model alongside the available data from experiments in humans and estimate the model parameters. In order to achieve this aim we linearize the model and use several methods of model and parameter validation: stability analysis, structural identifiability analysis, and sensitivity analysis based on traditional sensitivity functions and generalized sensitivity functions. We find that all model parameters are identifiable, structurally and taking into account parameter correlations, when several types of model output are used for parameter estimation. Based on these analyses we estimate parameter values from the limited available data and compare them with previously published parameter values. Finally we show how the model can be applied in future studies of treatment effectiveness in IgG multiple myeloma with simulations of serum monoclonal IgG responses during treatment.

## 1. Introduction

Immunoglobulin G (IgG) is protected from degradation by the neonatal Fc receptor (FcRn), resulting in an unusually long metabolic half-life at normal concentrations (~23 days; Rosenthal and Tan, 2010) and a high serum concentration in healthy adults (10–16 g l^−1^; Hall and Yates, 2010). The half-life of IgG is not constant, but varies with its serum concentration, due to saturation of recycling receptors. Elevated IgG concentrations saturate receptors such that a greater proportion of circulating IgG is degraded; conversely at low concentrations a greater proportion of IgG is recycled and the half-life is extended. Circulating IgG is internalized into intracellular endosomes in order to be degraded. FcRn expressed within the cells binds IgG inside the acidic environment of endosomes with a pH-dependent affinity. FcRn then sequesters the bound IgG away from the degradation pathway and back to the cell membrane, releasing it once again into the circulation. Those IgG molecules that are not bound to FcRn continue to follow the pathway to be degraded in lysosomes (Junghans and Anderson, 1996).

In multiple myeloma, clonal plasma cells in the bone marrow secrete a unique, monoclonal immunoglobulin (Ig). Half of patients have IgG-producing clones and are said to have IgG myeloma (Anderson, 2003). The monoclonal Ig produced by the cancer offers a convenient opportunity for clinicians to monitor the response of the tumor to therapy via the secreted protein, which is readily quantified in a blood sample. The cancer itself is only accessible by bone marrow biopsy or aspirate, both of which are unpleasant, invasive procedures. The concentration of monoclonal Ig in the blood is therefore the preferred measure by which the tumor is monitored; patient monitoring in clinical trials and the non-trial setting alike is heavily reliant on measurements of monoclonal Ig concentration in the blood (Kumar et al., 2016).

IgG myeloma patients typically present with an elevated concentration of serum monoclonal IgG. During treatment, the malignant plasma cells are killed and the production rate of monoclonal IgG correspondingly decreases, resulting in a fall in serum monoclonal IgG concentration. In this way, the serum monoclonal IgG response is used as a surrogate for the tumor response to treatment. The possible effects of the metabolism of IgG on its application as a cancer marker in multiple myeloma have been little studied, but are acknowledged in the literature. Sullivan and Salmon (1972) first brought the issue of IgG metabolism to the attention of the multiple myeloma community. Serum monoclonal IgG concentration, plasma volume, and IgG synthesis rate per cell were measured in 11 patients with IgG myeloma. Calculating the fractional catabolic rate of IgG using the equation provided by Waldmann and Strober (1969), Sullivan and Salmon (1972) estimated the tumor burden at a number of time points during treatment for each patient, concluding that increases and decreases in the tumor burden were underestimated by increases and decreases in monoclonal IgG. More recently, Bradwell et al. (2013), Koulieris et al. (2012), and Durie et al. (2006) have cited the concentration-dependent metabolism of IgG as a possible explanation for why monoclonal IgG may be seen as an unreliable response marker in multiple myeloma.

In order to investigate the dynamics of IgG in multiple myeloma, a mathematical model characterizing the metabolism of endogenous IgG in humans is required. Many mathematical models of IgG metabolism have been published in the literature (more than 20 at the time of writing), usually with the aim of describing the pharmacokinetics of therapeutic monoclonal antibodies that are similarly regulated by FcRn. Many of the models are therefore pharmacokinetic in nature: their parameter values are obtained from animal experiments and they may be physiologically based, with up to ten organs and the lymphatic system explicitly represented in the model (Hansen and Balthasar, 2003; Ferl et al., 2005; Garg and Balthasar, 2007; Fang and Sun, 2008; Urva et al., 2010; Chen and Balthasar, 2012; Deng et al., 2012; Xiao, 2012; Yan et al., 2012; Fronton et al., 2014; Ng et al., 2014). Physiologically based pharmacokinetic (PBPK) models may be unnecessarily complex for investigating serum IgG dynamics in multiple myeloma, particularly considering the limited human-derived data that are available for parameter estimation. More suitably, several authors have proposed a comparatively simple two-compartment model of IgG metabolism in which saturable recycling by FcRn is described using Michaelis–Menten kinetics (Waldmann and Strober, 1969; Kim et al., 2007; Hattersley et al., 2013). They also provide certain parameter values for humans.

In order to investigate serum IgG responses in IgG multiple myeloma, the parameter values used are highly important in order to have confidence in model-based predictions. Parameter estimation using limited data is an important problem in the mathematical modeling of physiological systems. Methods for parameter identification including structural identifiability analysis and sensitivity analysis should be used in the early stages of the model validation process; specifically, these analyses address whether parameters can be estimated from the available measurements and, where further experiments are possible, inform experiment design. In this paper we analyse the nonlinear two-compartment model of IgG metabolism (Waldmann and Strober, 1969; Kim et al., 2007; Hattersley et al., 2013) and the available measurements in humans for structural identifiability and sensitivity, in order to make optimal use of the limited data available in the literature. Having considered the identifiability problem, we estimate parameter values from the data, with the intention that the model can be used in the future to make generalized predictions for patients.

## 2. Methods

### 2.1. Experimental data

Data for parameter estimation were obtained from the literature. Studies of protein metabolism involve intravenously injecting a subject with radioisotopically labeled protein, known as a tracer, and then monitoring the proportion of tracer remaining in the blood over a period of time following administration. Radioactive tracers allow for distinction between the injected dose and the endogenously produced protein, enabling direct visualization of the distribution and elimination processes of a protein despite it being homeostatic. The radioactive label (usually iodine) remains bound to the protein until the protein is degraded, at which point the label is released and rapidly excreted in urine. Several tracer studies were performed for IgG in humans in the middle of the last century and the results collated by Waldmann and Strober (1969).

#### 2.1.1. Individual timecourse data

**Figure 1:**
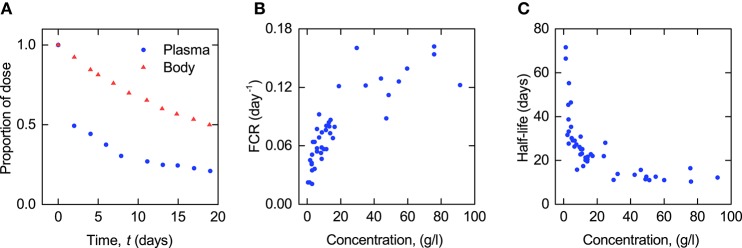
**(A)** Proportion of administered IgG remaining in plasma (blue circles) and the body (red triangles) in a typical normal subject; data from Solomon et al. (1963). Plasma concentration dependence of **(B)** fractional catabolic rate (FCR) and **(C)** half-life (*T*_½_) of IgG; redrawn from Waldmann and Strober (1969) with permission from S. Karger AG, Basel.

The data from a single subject consist of the timecourse of the proportion of an administered dose of radiolabeled IgG remaining in plasma and the proportion remaining in the whole body, calculated by subtracting the radioactivity in urine from the administered dose. The data collected from an individual are shown in Figure 1A. The data have been extracted from a plot by Solomon et al. (1963) using OriginPro^1^. Seven plots of this type have been found by the authors in the literature. The data in these plots are assumed to arise from seven individuals to whom we refer as subjects A–G. The data for subjects A–D are taken from Solomon et al. (1963), for subjects E and F from Waldmann and Terry (1990) and for subject G from Waldmann and Strober (1969). Subjects A and C have IgG myeloma, subject D has macroglobulinemia and subject E has familial hypercatabolic hypoproteinemia. These conditions do not preclude the subjects from this study but there may be a correlation between these conditions and individuals' parameter values of IgG metabolism; this is discussed in Section 3.2.

#### 2.1.2. Fractional catabolic rate and half-life

In compartmental analysis, parameters are often considered as either micro constants or macro (hybrid) constants. The micro constants are dependent upon the assumed structure of the compartmental model, whereas the macro constants can be determined directly from the profile of concentration or radioactivity over time, such as the exponents of a multi-exponential profile, and do not assume a particular model structure (Riviere, 2011).

Waldmann and Strober (1969) have plotted two macro parameters, the fractional catabolic rate (FCR) and the terminal half-life (*T*_½_), that can be calculated directly from an individual subject's timecourse of radioactivity. The FCR is defined as the fraction of the administered IgG in plasma that is catabolized per day and is calculated by dividing the rate at which the administered dose leaves the body at any time *t* > 0 by the amount of the dose remaining in plasma at that time. The rate at which the dose leaves the body is given by the slope of the timecourse of the dose remaining in the whole body. The *T*_½_ is defined as the time taken for half of the administered IgG to be eliminated, after completion of the distribution phase. This is obtained from the terminal slope of the timecourse observations plotted on a logarithmic scale.

The plots of FCR and *T*_½_ provided by Waldmann and Strober (1969) are reproduced in Figures 1B,C. Each point in these plots was obtained from the timecourse data of a single subject, an example of which is shown in Figure 1A. The parameters have been taken from a large number of subjects (FCR − 41 subjects; *T*_½_ − 44 subjects) with a wide range of plasma concentrations of IgG, in order to capture the concentration-dependent behavior of IgG metabolism. Macro parameters are functions of the micro parameters of the assumed compartmental structure—therefore in this paper the FCR and *T*_½_ data are used in the estimation of the parameters of the underlying compartmental model.

### 2.2. Model of endogenous IgG metabolism

**Figure 2:**
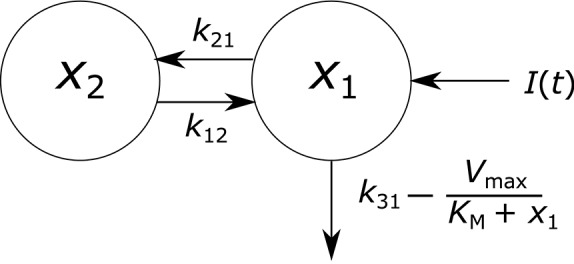
**Endogenous IgG metabolism model schematic**.

The nonlinear two-compartment model of endogenous IgG metabolism, with Michaelis–Menten kinetics describing the rate of recycling by FcRn receptors (Waldmann and Strober, 1969; Kim et al., 2007; Hattersley et al., 2013), is given by:

(1) $$\begin{array}{l} {\dot{x}}_{1}(t)=-\left(k_{21}+k_{31}-\frac{V_{\text{max}}}{K_{\text{M}}+x_{1}(t)}\right)x_{1}(t)+k_{12}x_{2}(t)+I(t)\\ {\dot{x}}_{2}(t)=k_{21}x_{1}(t)-k_{12}x_{2}(t) \end{array}$$

where *x*_1_(*t*) and *x*_2_(*t*) represent the quantities in µmol of IgG in plasma and in a peripheral compartment, respectively. *I*(*t*) represents the synthesis of IgG into plasma in µmol day^−1^. Rate constants *k*_*ij*_ represent material flow from compartment *j* to compartment *i*. The rate constant of the removal of IgG from the plasma compartment into intracellular endosomes for degradation is given by *k*_31_, with the indices denoting the transfer from plasma to a third compartment representing intracellular endosomes, which is omitted from the model. The rate of FcRn-mediated recycling, as a fraction of the quantity of IgG in plasma, is given by *V*_max_/(*K*_M_ + *x*_1_(*t*)). The parameters *V*_max_ and *K*_M_ are the maximum absolute rate of FcRn-mediated recycling in µmol day^−1^ and the Michaelis constant, representing the quantity of IgG in plasma in µmol at which the absolute recycling rate is half *V*_max_. Those IgG molecules which are removed from the plasma compartment into intracellular endosomes and which do not get recycled by FcRn are degraded in lysosomes. The amino acid products of lysosomal degradation are reused in the synthesis of new proteins (Appelqvist et al., 2013). A schematic of the system model is shown in Figure 2. Table 1 summarizes the model states and parameters.

**Table 1:** **States and parameters of IgG metabolism model**.

**Name**	**Units**	**Physiological interpretation**
*x*_1_	µmol	Quantity of IgG in the central (plasma) compartment
*x*_2_	µmol	Quantity of IgG in the peripheral (tissue) compartment
*k*_21_	day^−1^	Rate constant of flow of IgG from plasma to peripheral compartment
*k*_31_	day^−1^	Rate constant of flow of IgG from plasma into endosomes by pinocytosis
*k*_12_	day^−1^	Rate constant of flow of IgG from peripheral compartment to plasma
*V*_max_	µmol day^−1^	Maximum absolute recycling rate
*K*_M_	µmol	Michaelis constant; the quantity of IgG in plasma at which the absolute recycling rate is half *V*_max_

All states and parameters can only take non-negative values. The rate at which IgG is recycled cannot exceed the rate at which it leaves the plasma compartment to be degraded in intracellular endosomes; equivalently, the net elimination rate must be positive for all states and input rates: $k_{31}-\frac{V_{\text{max}}}{K_{\text{M}}}>0$.

When the production rate of IgG is assumed constant, *I*(*t*) = *I*_0_, in order to determine the model's steady states, solving $\dot{x}$_1_(*t*) = 0 and $\dot{x}$_2_(*t*) = 0 simultaneously gives the equilibrium point:

(2) $$\begin{array}{l} {\hat{x}}_{1}=\frac{-k_{31}K_{\text{M}}+I_{0}+V_{\text{max}}+\sqrt{4k_{31}K_{\text{M}}I_{0}+{\left(-k_{31}K_{\text{M}}+I_{0}+V_{\text{max}}\right)}^{2}}}{2k_{31}}\\ {\hat{x}}_{2}=\frac{k_{21}}{k_{12}}{\hat{x}}_{1}. \end{array}$$

The stability of this equilibrium point for all parameter values is demonstrated in Section 2.2.1.

#### 2.2.1. Stability of steady states

Linearizing the system described by Equation (1) about the equilibrium point gives:

(3) $$\left(\begin{array}{c} {\dot{x}}_{1}(t)\\ {\dot{x}}_{2}(t) \end{array}\right)=\left(\begin{array}{cc} -k_{21}-k_{31}-\frac{V_{\text{max}}{\hat{x}}_{1}}{{\left(K_{\text{M}}+{\hat{x}}_{1}\right)}^{2}} & k_{12}\\ k_{21} & -k_{12} \end{array}\right)\left(\begin{array}{c} x_{1}(t)\\ x_{2}(t) \end{array}\right).$$

According to the Routh–Hurwitz stability criterion, the two-state system is stable provided the coefficients of the characteristic polynomial of the linearized system are positive (Routh, 1877). The coefficients of the characteristic polynomial are given by:

(4) $$\begin{array}{l} a_{2}=1\\ a_{1}=\frac{\begin{array}{l} k_{31}K_{\text{M}}^{2}-K_{\text{M}}V_{\text{max}}+2k_{31}K_{\text{M}}{\hat{x}}_{1}+k_{31}{\hat{x}}_{1}^{2}+k_{12}{\left(K_{\text{M}}+{\hat{x}}_{1}\right)}^{2}\\ \;+\;k_{21}{\left(K_{\text{M}}+{\hat{x}}_{1}\right)}^{2} \end{array}}{{\left(K_{\text{M}}+{\hat{x}}_{1}\right)}^{2}}\\ a_{0}=\frac{k_{12}\left(k_{31}{\left(K_{\text{M}}+{\hat{x}}_{1}\right)}^{2}-K_{\text{M}}V_{\text{max}}\right)}{{\left(K_{\text{M}}+{\hat{x}}_{1}\right)}^{2}}. \end{array}$$

The denominators in the expressions for *a*_0_ and *a*_1_ are always positive. All parameters and the steady state ${\hat{x}}_{1}$ are positive. The sign of *a*_0_ is thus given by the sign of $\left(k_{31}{\left(K_{\text{M}}+{\hat{x}}_{1}\right)}^{2}-K_{\text{M}}V_{\text{max}}\right)$. For stability of the equilibrium point it is necessary that $\left(k_{31}{\left(K_{\text{M}}+{\hat{x}}_{1}\right)}^{2}-K_{\text{M}}V_{\text{max}}\right)>0$. This condition is met when $k_{31}K_{\text{M}}^{2}-K_{\text{M}}V_{\text{max}}>0$, or equivalently $k_{31}-\frac{V_{\text{max}}}{K_{\text{M}}}>0$. The sign of *a*_1_ is given by the sign of its numerator, $k_{31}K_{\text{M}}^{2}-K_{\text{M}}V_{\text{max}}+2k_{31}K_{\text{M}}{\hat{x}}_{1}+k_{31}{\hat{x}}_{1}^{2}+k_{12}{\left(K_{\text{M}}+{\hat{x}}_{1}\right)}^{2}+k_{21}{\left(K_{\text{M}}+{\hat{x}}_{1}\right)}^{2}$. Once again, the sign of *a*_1_ is positive provided that $k_{31}-\frac{V_{\text{max}}}{K_{\text{M}}}>0$.

Both of the coefficients *a*_0_ and *a*_1_ are positive provided that all parameter values are positive and $k_{31}-\frac{V_{\text{max}}}{K_{\text{M}}}>0$. Referring back to Equation (1), that is the condition which ensures a positive IgG elimination rate for all *x*_1_ > 0. A negative elimination rate does not make sense physiologically and as such parameter values are not permitted which violate this condition. The equilibrium point is thus stable for all permitted parameter values.

### 2.3. Model of observed measurements

In this section we consider how the observable measurements (timecourse of radioactivity, FCR and *T*_½_) relate to the system model. Tracer experiments are designed specifically so that the tracer-labeled protein observes linear kinetics, despite the mode of metabolism being in fact nonlinear (Anderson, 1983). A linear model describing the timecourse observations is derived here.

#### 2.3.1. Timecourse observations

Assuming that the radiolabeled IgG dose and unlabeled endogenous IgG are indistinguishable by the system, both are described by the model in Equation (1). The injected and endogenous IgG can be explicitly represented by letting *x*_*i*_(*t*) = *x*_*i*, T_(*t*) + *x*_*i*, E_(*t*) for *i* = 1, 2, with “T” denoting tracer and “E” denoting endogenous IgG. Then, from Equation (1), the dynamics of labeled and unlabeled IgG are given by:

(5) $$\begin{array}{l} {\dot{x}}_{1,\text{T}}(t)=-\left(k_{21}+k_{31}-\frac{V_{\text{max}}}{K_{\text{M}}+x_{1,\text{E}}(t)+x_{1,\text{T}}(t)}\right)x_{1,\text{T}}(t)\\ \;+\;k_{12}x_{2,\text{T}}(t)\\ {\dot{x}}_{2,\text{T}}(t)=k_{21}x_{1,\text{T}}(t)-k_{12}x_{2,\text{T}}(t)\\ {\dot{x}}_{1,\text{E}}(t)=-\left(k_{21}+k_{31}-\frac{V_{\text{max}}}{K_{\text{M}}+x_{1,\text{E}}(t)+x_{1,\text{T}}(t)}\right)x_{1,\text{E}}(t)\\ \;+\;k_{12}x_{2,\text{E}}(t)+I_{\text{E}}\\ {\dot{x}}_{2,\text{E}}(t)=k_{21}x_{1,\text{E}}(t)-k_{12}x_{2,\text{E}}(t) \end{array}$$

where *x*_*i*, T_(*t*) and *x*_*i*, E_(*t*) represent the quantities in µmol of radiolabeled and endogenous IgG in compartment *i*, respectively.

The intravenous bolus injection of tracer can be treated as a non-zero initial condition for *x*_1,T_(*t*); thus the initial conditions of the tracer are given by:

(6) $$\begin{array}{l} x_{1,\text{T}}(0)=D\\ x_{2,\text{T}}(0)=0 \end{array}$$

where *D* is the dose of tracer in µmol. The production rate of endogenous IgG, *I*_E_ µmol day^−1^, is assumed constant. The initial conditions of the endogenous IgG are given by the equilibrium point in Section 2.2, with *I*_0_ = *I*_E_. The experimenter measures the proportion of the initially injected radioactivity in plasma and in the whole body. The observation functions are thus given by:

(7) $$\begin{array}{l} y_{1}(t)=x_{1,\text{T}}(t)/D\\ y_{2}(t)=\left(x_{1,\text{T}}(t)+x_{2,\text{T}}(t)\right)/D. \end{array}$$

A sufficiently small quantity of radiolabeled IgG, typically 0.5–1 mg (3.33 × 10^−3^–6.67 × 10^−3^ µmol) (Solomon et al., 1963), is administered into plasma so as not to perturb the steady state of the endogenous protein. Thus *x*_1,E_ and *x*_2,E_ can be assumed constant. Then the equations describing the tracer dynamics are no longer coupled with those describing the endogenous IgG dynamics. A second assumption is required in order to derive a linear model: the quantity of tracer, *x*_1,T_(*t*), is assumed to be much smaller than the quantity of the subject's endogenous IgG, *x*_1,E_. Thus the term $\frac{V_{\text{max}}}{K_{\text{M}}+x_{1,\text{E}}+x_{1,\text{T}}\left(t\right)}$ can be approximated by $\frac{V_{\text{max}}}{K_{\text{M}}+x_{1,\text{E}}}$. In this way, the elimination rate of the tracer is determined by the quantity of the subject's endogenous plasma IgG only. A further simplification can be made by noticing that for a linear model, the initial conditions and observation gain cancel out (see Equations 6, 7). The equations describing the tracer kinetics are thus given by:

(8) $$\begin{array}{l} {\dot{x}}_{1,\text{P}}(t)=-\left(k_{21}+k_{31}-\frac{V_{\text{max}}}{K_{\text{M}}+x_{1,\text{E}}}\right)x_{1,\text{P}}(t)+k_{12}x_{2,\text{P}}(t)\\ {\dot{x}}_{2,\text{P}}(t)=k_{21}x_{1,\text{P}}(t)-k_{12}x_{2,\text{P}}(t) \end{array}$$

where *x*_1,P_(*t*) and *x*_2,P_(*t*) represent the proportion of the radiolabeled IgG dose *D* in the central and peripheral compartments, respectively, at time *t*. *x*_1,E_ represents the quantity of the subject's endogenous IgG in the central compartment, which is assumed to remain in steady state. All other parameters are defined as in Section 2.2.

The initial conditions of the model are now given by:

(9) $$\begin{array}{l} x_{1,\text{P}}(0)=1\\ x_{2,\text{P}}(0)=0. \end{array}$$

The corresponding observation functions are given by:

(10) $$\begin{array}{l} y_{1}(t)=x_{1,\text{P}}(t)\\ y_{2}(t)=x_{1,\text{P}}(t)+x_{2,\text{P}}(t). \end{array}$$

**Figure 3:**
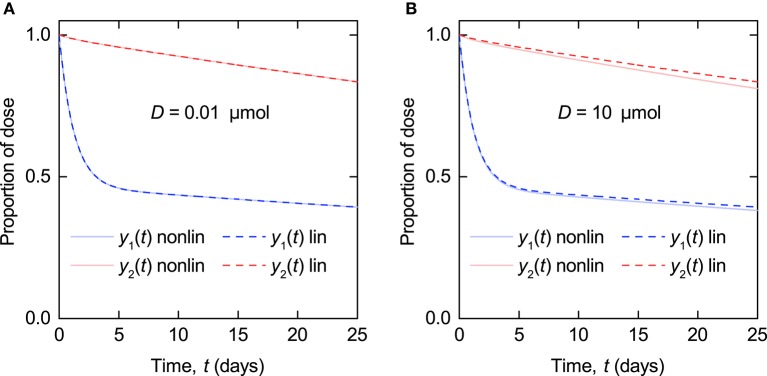
**Simulations of timecourse responses ***y***_**1**_(***t***) and ***y***_**2**_(***t***) as described by Equations (5–7) (nonlinear model – solid line) and Equations (8–10) (linearized model – dashed line)**. The quantity of endogenous IgG in plasma at *t* = 0, *x*_1,E_(0), is 5 µmol. The tracer dose *D* is **(A)** 0.01 µmol and **(B)** 10 µmol.

The linearized model represented by Equations (8–10) is a valid approximation of the nonlinear model (Equations 5–7) when the administered dose of radiolabeled IgG, *D*, is sufficiently smaller than the quantity of endogenous IgG in plasma at *t* = 0, *x*_1,E_(0). In Figure 3 simulations of the nonlinear model and the linearized model are compared. The parameter values used are those estimated in this paper and summarized in **Table 6**. The production rate of endogenous IgG is set to *I*_E_ = 0.0727 µmol day^−1^ to give *x*_1,E_(0) = 5 µmol for the nonlinear model and *x*_1,E_ = 5 µmol for the linearized model, representing the lower limit of the quantities of endogenous IgG in plasma seen in the data. In Figure 3A the tracer dose *D* is 0.01 µmol, representing the upper limit of administered tracer doses (Solomon et al., 1963). The nonlinear and linearized model responses are indistinguishable, illustrating that for typical tracer doses the linearized model is a valid approximation of the nonlinear model. In Figure 3B the tracer dose *D* is 10 µmol, 1,000 times larger; at this point the assumptions weaken and there is a noticeable difference between the responses of the two models.

#### 2.3.2. Fractional catabolic rate and half-life

The FCR is defined as the proportion of the radiolabeled IgG in plasma that is catabolized per day. From Equation (8) this is given by:

(11) $$\text{FCR}=k_{31}-\frac{V_{\text{max}}}{K_{\text{M}}+x_{1,\text{E}}}.$$

The terminal half-life, *T*_½_, is related to the elimination phase of the kinetics, after the distribution phase is complete. The model described by Equation (8) is a linear two-compartment model with the solutions for *x*_1,P_(*t*) and *x*_2,P_(*t*) given by the bi-exponential functions:

(12) $$\begin{array}{l} x_{1,\text{P}}(t)=A_{11}\text{exp}(\lambda_{1}t)+A_{12}\text{exp}(\lambda_{2}t)\\ x_{2,\text{P}}(t)=A_{21}\text{exp}(\lambda_{1}t)+A_{22}\text{exp}(\lambda_{2}t) \end{array}$$

where *A*_*ij*_ and λ_*j*_ are macro constants, with |λ_1_| > |λ_2_|. By definition, *T*_½_ is given by:

(13) $$T_{½}=-\frac{\text{log }2}{\lambda_{2}}.$$

Solving Equation (8) for λ_2_ and substituting into Equation (13) gives the following expression for *T*_½_ in terms of the micro parameters of the model:

(14) $$\begin{array}{l} \;T_{½}=2\text{ log }2/\;(k_{12}+k_{21}+k_{31}-\frac{V_{\text{max}}}{K_{\text{M}}+x_{1,\text{E}}}\\ -\;\sqrt{-4k_{12}\left(k_{31}-\frac{V_{\text{max}}}{K_{\text{M}}+x_{1,\text{E}}}\right)+{\left(k_{12}+k_{21}+k_{31}-\frac{V_{\text{max}}}{K_{\text{M}}+x_{1,\text{E}}}\right)}^{2})}. \end{array}$$

From Equations (11, 14), we find that the relationship between *T*_½_ and FCR is given by:

(15) $$T_{½}=\frac{2\text{log}2}{k_{12}+k_{21}+\text{FCR}-\sqrt{-4k_{12}\text{FCR}+{\left(k_{12}+k_{21}+\text{FCR}\right)}^{2}}}.$$

## 3. Results

### 3.1. Structural identifiability of model parameters

Structural identifiability addresses the question of whether model parameters can be uniquely identified from available observations, under the assumption of the availability of ideal (i.e., noise-free) and continuous observational data. Structural identifiability of parameters does not imply that they are identifiable in practice, from observations that are inevitably measured with noise; therefore in this paper structural identifiability analysis is used alongside sensitivity analysis.

Here we determine which of the model parameters are structurally uniquely identifiable from the following measurements: an individual subject's timecourse, FCR vs. the quantity of endogenous IgG in plasma, and *T*_½_ vs. the quantity of endogenous IgG in plasma.

#### 3.1.1. Individual timecourse

Here the transfer function method is used (Bellman and Åström, 1970). To apply this approach the system described by Equations (8–10) is re-written in vector-matrix notation as

(16) $$\begin{array}{l} \dot{x}(t,p)=A(p)x(t,p)+B(p)u(t)\\ x(0,p)=0\\ y(t,p)=C(p)x(t,p), \end{array}$$

where *x*(*t, p*) = (*x*_1,P_(*t*), *x*_2,P_(*t*)), and *y*(*t, p*) = (*y*_1_(*t*), *y*_2_(*t*)) are column vectors representing the state and the observation, respectively. *u*(*t*) represents the single input to the system, an impulse at time *t* = 0, given by *u*(*t*) = δ(*t*). *A*(*p*) and *C*(*p*) are 2 × 2 matrices and *B*(*p*) is a column vector. *A*(*p*), *B*(*p*), and *C*(*p*) are given by:

(17) $$\begin{array}{l} A(p)=\left(\begin{array}{cc} -\left(k_{21}+k_{31}-\frac{V_{\text{max}}}{K_{\text{M}}+x_{1,\text{E}}}\right) & k_{12}\\ k_{21} & -k_{12} \end{array}\right),\\ B(p)=\left(\begin{array}{c} 1\\ 0 \end{array}\right),\;C(p)=\left(\begin{array}{cc} 1 & 0\\ 1 & 1 \end{array}\right). \end{array}$$

Note that the administration of a bolus dose is now represented as an impulse at time *t* = 0, rather than a non-zero initial condition, such that *x*(0, *p*) = 0.

Taking Laplace transforms of Equation (16), the input-output relation is described by *Y*(*s*) = *G*(*s*)*U*(*s*), where *G*(*s*) is the transfer function matrix, given by *G*(*s*) = *C*(*p*)(*sI* − *A*(*p*))^−1^*B*(*p*), where *I* is the 2 × 2 identity matrix. *G*(*s*) has two elements, corresponding to the two measured outputs, which are given by:

(18) $$\begin{array}{l} G_{1}(s)=\frac{s\;+\;k_{12}}{\begin{array}{c} s^{2}\;+\;\left(k_{31}\text{​ }-\text{ ​}\frac{V_{\text{max}}}{K_{\text{M}}+x_{1,\text{E}}}\;+\;k_{12}\;+\;k_{21}\right)\\ s\;+\;\left(k_{31}\;-\;\frac{V_{\text{max}}}{K_{\text{M}}+x_{1,\text{E}}}\right)k_{12} \end{array}}\\ G_{2}(s)=\frac{s\;+\;k_{12}\;+\;k_{21}}{\begin{array}{c} s^{2}\;+\;\left(k_{31}\;-\;\frac{V_{\text{max}}}{K_{\text{M}}+x_{1,\text{E}}}\;+\;k_{12}\;+\;k_{21}\right)\\ s\;+\;\left(k_{31}\;-\;\frac{V_{\text{max}}}{K_{\text{M}}+x_{1,\text{E}}}\right)k_{12}. \end{array}} \end{array}$$

Let Φ(*p*) = (ϕ_1_(*p*), …, ϕ_4_(*p*)), where *p* = (*k*_12_, *k*_21_, *k*_31_, *V*_max_, *K*_M_, *x*_1,E_), denote the (distinct) coefficients of *s* in Equation (18). The coefficients, Φ(*p*), are uniquely determinable from the input-output relationship of the system and are given by:

(19) $$\begin{array}{l} \phi_{1}(p)=k_{12}\\ \phi_{2}(p)=k_{12}+k_{21}\\ \phi_{3}(p)=k_{12}\left(k_{31}-\frac{V_{\text{max}}}{K_{\text{M}}+x_{1,\text{E}}}\right)\\ \phi_{4}(p)=k_{31}-\frac{V_{\text{max}}}{K_{\text{M}}+x_{1,\text{E}}}+k_{12}+k_{21}. \end{array}$$

Introducing an alternative parameter vector $\overset{-}{p}=\left({\overset{-}{k}}_{12},{\overset{-}{k}}_{21},{\overset{-}{k}}_{31},{\overset{-}{V}}_{\text{max}},{\overset{-}{K}}_{\text{M}},{\overset{-}{x}}_{1,\text{E}}\right)$ and equating $\Phi \left(p\right)=\Phi \left(\overset{-}{p}\right)$, it can readily be seen from ϕ_1_(*p*) and ϕ_2_(*p*) that the parameters *k*_12_ and *k*_21_ are uniquely determined (i.e., $k_{12}={\overset{-}{k}}_{12}$ and $k_{21}={\overset{-}{k}}_{21}$) and therefore structurally globally identifiable from the timecourse of radioactivity remaining in plasma and the body. The parameters *k*_31_, *V*_max_, *K*_M_ and *x*_1,E_ are not uniquely identifiable; however the FCR (given by Equation 11) is uniquely identifiable.

#### 3.1.2. Fractional catabolic rate

The relationship between the FCR and *x*_1,E_ is given by Equation (11). The SolveAlways function was used in Mathematica^2^ to find out whether the parameters *k*_31_, *V*_max_, and *K*_M_ are uniquely determinable by the relationship in Equation (11). Introducing an alternative parameter vector $\left({\overset{-}{k}}_{31},{\overset{-}{V}}_{\text{max}},{\overset{-}{K}}_{\text{M}}\right)$ and solving the equation:

(20) $$k_{31}-\frac{V_{\text{max}}}{K_{\text{M}}+x_{1,\text{E}}}={\bar{k}}_{31}-\frac{{\bar{V}}_{\text{max}}}{{\bar{K}}_{\text{M}}+x_{1,\text{E}}},$$

over all values of *x*_1,E_, gives $\left({\overset{-}{k}}_{31},{\overset{-}{V}}_{\text{max}},{\overset{-}{K}}_{\text{M}}\right)=\left(k_{31},V_{\text{max}},K_{\text{M}}\right)$ as the only solution for the unknown parameters. Therefore, the parameters *k*_31_, *V*_max_, and *K*_M_ are uniquely determinable from the relationship between the FCR and *x*_1,E_.

#### 3.1.3. Terminal half-life

The relationship between *T*_½_ and *x*_1,E_ is given by Equation (14). We now wish to know whether the parameter vector *p* = (*k*_12_, *k*_21_, *k*_31_, *V*_max_, *K*_M_) is uniquely determinable from the relationship in Equation (14). From Equation (13), this is equivalent to asking whether *p* is uniquely determinable from the relationship between λ_2_ and *x*_1,E_, given by:

(21) $$\begin{array}{l} \lambda_{2}=\frac{1}{2}(-k_{12}-k_{21}-k_{31}+\frac{V_{\text{max}}}{K_{\text{M}}+x_{1,\text{E}}}\\ +\;\sqrt{-4k_{12}\left(k_{31}\text{​}-\text{​}\frac{V_{\text{max}}}{K_{\text{M}}+x_{1,\text{E}}}\right)\text{​ }+\text{ ​}{\left(k_{12}+k_{21}+k_{31}-\frac{V_{\text{max}}}{K_{\text{M}}+x_{1,\text{E}}}\right)}^{2})}. \end{array}$$

The structural identifiability problem amounts to determining whether there exists an alternative parameter vector $\overset{-}{p}$ such that $\lambda_{2}\left(x_{1,\text{E}},p\right)=\lambda_{2}\left(x_{1,\text{E}},\overset{-}{p}\right)$ with $p\neq \overset{-}{p}$. λ_2_ is one of the roots of the characteristic polynomial equation, given by:

(22) $$\begin{array}{l} \lambda^{2}+\left(k_{12}+k_{21}+k_{31}-\frac{V_{\text{max}}}{K_{\text{M}}+x_{1,\text{E}}}\right)\lambda \\ \;+\;k_{12}\left(k_{31}-\frac{V_{\text{max}}}{K_{\text{M}}+x_{1,\text{E}}}\right)=0. \end{array}$$

We wish to know whether there exists an alternative parameter vector $\overset{-}{p}\neq p$, such that:

(23) $$\begin{array}{l} \lambda^{2}+\left(k_{12}+k_{21}+k_{31}-\frac{V_{\text{max}}}{K_{\text{M}}+x_{1,\text{E}}}\right)\lambda +k_{12}\left(k_{31}-\frac{V_{\text{max}}}{K_{\text{M}}+x_{1,\text{E}}}\right)\\ \;=\lambda^{2}+\left({\bar{k}}_{12}+{\bar{k}}_{21}+{\bar{k}}_{31}-\frac{{\bar{V}}_{\text{max}}}{{\bar{K}}_{\text{M}}+x_{1,\text{E}}}\right)\lambda \\ \;+\;{\bar{k}}_{12}\left({\bar{k}}_{31}-\frac{{\bar{V}}_{\text{max}}}{{\bar{K}}_{\text{M}}+x_{1,\text{E}}}\right). \end{array}$$

The coefficients of the quadratic are unique, therefore

(24) $$\begin{array}{l} k_{12}+k_{21}+k_{31}-\frac{V_{\text{max}}}{K_{\text{M}}+x_{1,\text{E}}}={\bar{k}}_{12}+{\bar{k}}_{21}+{\bar{k}}_{31}-\frac{{\bar{V}}_{\text{max}}}{{\bar{K}}_{\text{M}}+x_{1,\text{E}}}\\ \;k_{12}\left(k_{31}-\frac{V_{\text{max}}}{K_{\text{M}}+x_{1,\text{E}}}\right)={\bar{k}}_{12}\left({\bar{k}}_{31}-\frac{{\bar{V}}_{\text{max}}}{{\bar{K}}_{\text{M}}+x_{1,\text{E}}}\right). \end{array}$$

The only solution to Equation (24), over all values of *x*_1,E_ and for positive parameter values only, is $p=\overset{-}{p}$. Therefore the parameters *k*_12_, *k*_21_, *k*_31_, *V*_max_, and *K*_M_ are uniquely determinable from the relationship between *T*_½_ and *x*_1,E_.

#### 3.1.4. Summary of structural identifiable parameters

From an individual's timecourse of radioactivity remaining in plasma and the body, described by Equations (8–10), the parameters *k*_12_ and *k*_21_ are structurally uniquely identifiable. The parameters *k*_31_, *V*_max_, *K*_M_, and *x*_1,E_ are not uniquely identifiable; however the FCR is uniquely identifiable. The parameter *x*_1,E_ may be measured independently; however the parameters *k*_31_, *V*_max_, and *K*_M_ remain unidentifiable even when *x*_1,E_ is known. This result is intuitive, as the unidentifiable parameters describe the nonlinear behavior which is not demonstrated when the quantity of endogenous IgG in plasma, *x*_1,E_, is constant.

In order to show the nonlinear behavior of the model, observations need to be made over a range of steady state quantities of endogenous IgG in plasma, *x*_1,E_. Given a set of timecourses, each described by Equations (8–10), for a range of different values of *x*_1,E_, it is possible to measure the FCR, *T*_½_ and *x*_1,E_ for each timecourse. From the relationship between the FCR and *x*_1,E_, the parameters *k*_31_, *V*_max_ and *K*_M_ are structurally globally identifiable. From the relationship between *T*_½_ and *x*_1,E_, the parameters *k*_12_, *k*_21_, *k*_31_, *V*_max_, and *K*_M_ are structurally globally identifiable. The structurally identifiable parameters are summarized in Table 2.

**Table 2:** **Structurally identifiable parameters**.

**Observation**	**Structurally globally identifiable parameters**
Individual subject's timecourse	*k*_12_, *k*_21_
FCR vs. *x*_1,E_	*k*_31_, *V*_max_, and *K*_M_
*T*_½_ vs. *x*_1,E_	*k*_12_, *k*_21_, *k*_31_, *V*_max_, and *K*_M_

### 3.2. Estimation of parameters from individual timecourse data

The model parameters *k*_12_ and *k*_21_ along with the FCR are structurally globally identifiable from the individual timecourse measurements *y*_1_(*t*) and *y*_2_(*t*), as described by the linearized model in Equations (8–10). These three parameters were estimated from timecourse data from seven individuals whom we refer to as subjects A–G. The data are described in Section 2.1.

**Figure 4:**
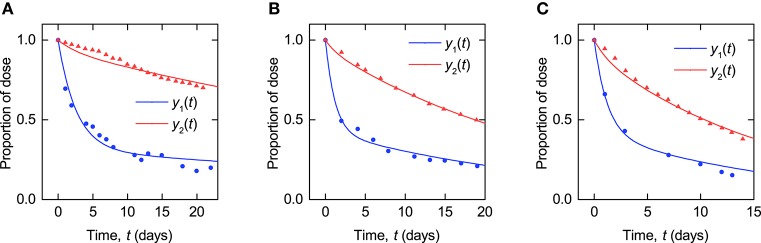
**Timecourse fits: model described by Equations (8–10) fitted to timecourse data extracted from plots in Solomon et al. (**1963**) for (A–C)** subjects A, B, and C.

Parameter values were estimated for each subject by analytically solving the linear ODE system and minimizing the sum of squared residual errors between the model output and the data, using the function NonlinearModelFit in Mathematica^2^. For each subject, the model outputs *y*_1_(*t*) and *y*_2_(*t*) were simultaneously fitted to the plasma and whole body timecourse data, respectively. Three examples of the fits (subjects A–C) are shown in Figure 4. The corresponding plots for all subjects are provided in the Supplementary Material.

**Figure 5:**
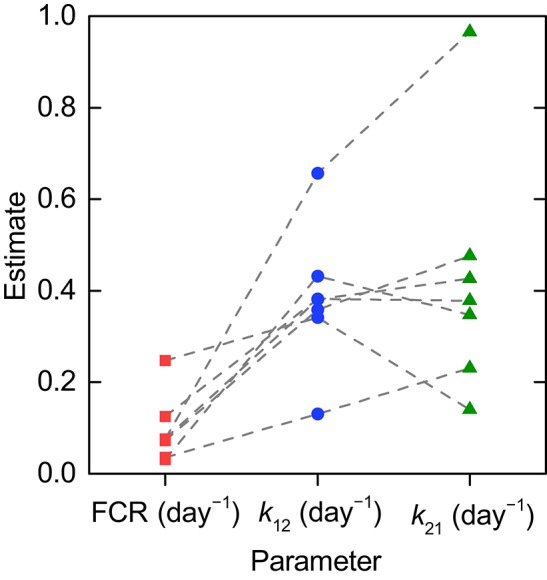
**Parameter estimates for individual timecourses**. Dashed lines connect the estimates obtained for an individual subject.

The parameter estimates and their standard errors are given in Table 3. For all three parameters across all subjects, the standard errors are small relative to their respective parameter estimates. The distribution of parameter estimates among the seven subjects is illustrated in Figure 5. The mean and median of each parameter are summarized in Table 3. The root mean squared error (RMSE), as a measure of the goodness-of-fit, for each fitted timecourse is also provided in Table 3.

**Table 3:** **Parameter estimates and their standard errors (SE) and the root mean squared error (RMSE) for each fitted timecourse**.

**Subject**	**FCR (day^−1^)**		***k*_12_ (day^−1^)**		***k*_21_ (day^−1^)**		**RMSE**
	**Estimate**	**SE**	**Estimate**	**SE**	**Estimate**	**SE**	
A	0.0359	0.00169	0.130	0.0182	0.231	0.0218	0.0336
B	0.0761	0.00190	0.381	0.0539	0.426	0.0546	0.0182
C	0.125	0.00397	0.382	0.0643	0.378	0.0516	0.0235
D	0.0311	0.000863	0.432	0.0746	0.347	0.0559	0.0136
E	0.247	0.00632	0.341	0.125	0.140	0.0333	0.0197
F	0.0728	0.00108	0.358	0.0233	0.476	0.0268	0.0134
G	0.0766	0.00149	0.656	0.0538	0.965	0.0716	0.0222
Mean	0.0950		0.383		0.423		
Median	0.0761		0.381		0.378		

As stated in Section 2.1, several subjects have IgG myeloma, macroglobulinemia or familial hypercatabolic hypoproteinemia. Patients with familial hypercatabolic hypoproteinemia do not express FcRn, explaining the large value of the FCR (0.247 day^−1^) for subject E. The parameter *V*_max_ (not estimated here) for subject E should be equal to zero, reflecting the absence of recycling receptors. Subjects A and C have IgG myeloma and subject D has macroglobulinemia. The high or low values of the FCR in these patients should be explained by the concentration-dependent catabolism of IgG, as described by Equation (11), with abnormally high or low plasma IgG concentrations likely occurring as symptoms of the respective disease.

#### 3.2.1. Sensitivity to model parameters

Along with structural identifiability, parameter identification requires sensitivity of the model output to the parameters. There are two types of sensitivity function: traditional sensitivity functions (TSFs) and generalized sensitivity functions (GSFs) (Thomaseth and Cobelli, 1999). Used together, TSFs and GSFs can provide insight in terms of the information about individual parameters provided by a measured output over the time duration of the experiment.

In order to estimate a model parameter from measurements of a model output it is necessary that the measured output is sensitive to the parameter over the time interval of the experiment (Banks et al., 2007). The TSF of a measured model output with respect to one component of the model parameter vector is given by the partial derivative of the output with respect to the parameter, for example:

(25) $$s_{\text{TSF},y_{1},k_{12}}(t)=\frac{\partial y_{1}}{\partial k_{12}}(t)$$

is the TSF of the model output *y*_1_(*t*) with respect to the parameter *k*_12_. The TSF is locally defined for the “true” parameter vector of the system. For the inverse problem in this section the true model and parameter vector for each subject is unknown; therefore the TSFs are calculated for the estimated parameter vectors for each subject to investigate parameter sensitivity over a range of parameter vectors that are likely to be seen in individuals.

**Figure 6:**
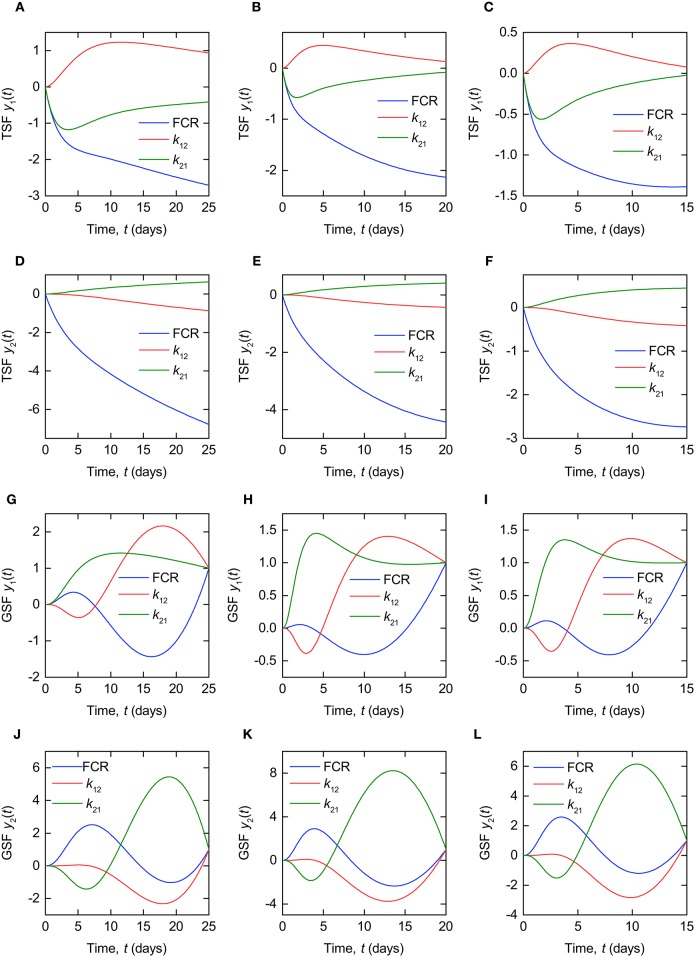
**Traditional sensitivity functions (TSFs) of timecourse outputs ***y***_**1**_(***t***), for (A–C)** subjects A, B and C, and *y*_2_(*t*), for **(D–F)** subjects A, B, and C. Generalized sensitivity functions (GSFs) of timecourse outputs *y*_1_(*t*), for **(G–I)** subjects A, B, and C, and *y*_2_(*t*), for **(J–L)** subjects A, B, and C.

The TSFs for the timecourse outputs *y*_1_(*t*) and *y*_2_(*t*) are plotted in Figure 6, evaluated at the parameter estimates for subjects A, B, and C, respectively, provided in Table 3. The TSFs were calculated in Mathematica^2^. The TSFs show that the model outputs are sensitive to all three parameters over the time interval of observation for each of the parameter vectors. The corresponding plots for all subjects are provided in the Supplementary Material. A similar pattern is observed for the remaining subjects D–G.

A shortcoming of the TSF is that it does not account for correlation between parameters. An alternative function, the GSF, takes account of parameter correlations and quantifies the information content of a model output on an individual parameter over the time duration of observation. The GSFs for a general model output function *y*(*t*) = *f*(*t*, θ_0_) with parameter vector θ_0_ are defined as:

(26) $$s_{\text{GSF},y,\theta }(t_{l})=\sum_{i\;=\;1}^{l}\frac{1}{\sigma^{2}(t_{i})}[F^{-1}\times \nabla_{\theta }f(t_{i},\theta_{0})]•\nabla_{\theta }f(t_{i},\theta_{0}),$$

where

(27) $$F=\sum_{j\;=\;1}^{n}\frac{1}{\sigma^{2}(t_{j})}\nabla_{\theta }f(t_{j},\theta_{0})\nabla_{\theta }f{\left(t_{j},\theta_{0}\right)}^{\text{T}}$$

is the Fisher information matrix and the model output *y*(*t*) = *f*(*t*, θ_0_) is observed with error at discrete times *t*_*l*_, *l* = 1, …, *n*. The measured values of the output are given by *Y*_*l*_ = *f*(*t*_*l*_, θ_0_) + ϵ_*l*_ with $\sigma^{2}\left(t_{l}\right)$ the variance of the error on the observation at time *t*_*l*_, ϵ_*l*_ (Thomaseth and Cobelli, 1999). In the definition of the GSF the true parameter vector θ_0_ is assumed known. Here the GSFs are calculated for the estimated parameter vectors for each subject, in order to investigate the inverse problem for the different dynamics seen in individuals.

The GSFs for the timecourse outputs *y*_1_(*t*) and *y*_2_(*t*) are plotted in Figure 6, for the parameter estimates of subjects A, B, and C, respectively, provided in Table 3. The GSFs were calculated in Mathematica^2^. Unlike the TSF, the GSF is defined only at discrete measurement times. In order to obtain an approximation of the smooth function for continuous measurement data, as in Thomaseth and Cobelli (1999) and Banks et al. (2007), we assume a high rate of sampling, calculating the GSF as though a measurement is taken every 0.1 days for each subject. We assume that the variances of the errors are equal at all measurement times, such that the variance terms cancel out in the definition of the GSF (see Equations 26, 27).

According to the interpretation of GSFs given by Thomaseth and Cobelli (1999), a steep increase in the GSF of a particular parameter between 0 and 1 indicates the interval on which the information on that parameter, provided by the measured output, is concentrated. Figures 6H,I show the ideal pattern of three distinct intervals of steep increase between 0 and 1 for the three parameters, respectively, with the information on *k*_21_ concentrated at the beginning of the experiment, followed by *k*_12_ and then the FCR. Intuitively this makes sense, because the dose is administered to the first compartment, from where it transfers to the second compartment in the initial days of the experiment. The pattern shown by the GSFs of *y*_1_(*t*) for subject A, depicted in Figure 6G, appears slightly different to that shown by subjects B and C; however, if we extend the experiment time to 50 days for subject A, the GSFs then appear extremely similar to those of subjects B and C. This is due to the slower dynamics exhibited by this subject—see Table 3 and Figure 4. The pattern exhibited by the GSFs of *y*_2_(*t*) for each patient indicates a high correlation between the parameters, producing large oscillations in the GSFs. The corresponding plots for all subjects are provided in the Supplementary Material.

The GSF is clearly a useful tool for understanding the behavior of estimators of correlated parameters; however, it is still important to use the TSF alongside the GSF when analysing parameter sensitivity. As mentioned, the GSFs of *y*_1_(*t*) for subject A appear more similar to those for subjects B and C when the duration of the experiment is extended. The trajectory of the GSF is dependent on the times at which data are collected, and is forced to equal one at the final measurement time. This can result in misleading GSFs when the observation interval is defined over a period of low sensitivity (as defined by the TSF) of the output to the parameters. For this reason we use the TSF and GSF alongside one another. This is discussed in more detail by Banks et al. (2007).

### 3.3. Estimation of parameters from fractional catabolic rate and half-life

As shown in Section 3.1, the parameters *k*_31_, *V*_max_, and *K*_M_ are structurally unidentifiable from an individual subject's timecourse data, assuming the linearized model given by Equations (8–10). It is therefore necessary to make use of the relationships between FCR and *T*_½_, respectively, and the quantity of endogenous IgG in plasma, *x*_1,E_. Unfortunately, these relationships are not known for an individual subject; obtaining them would require performing the tracer experiment over a range of different plasma concentrations of endogenous IgG within an individual subject, which is not practically feasible. We therefore estimate parameters from the FCR and *T*_½_ measurements taken from a sample of patients with a wide range of endogenous IgG plasma concentrations, as though the data arose from an individual subject, in what may be described as a naive pooled approach (Wright, 1998). It is therefore not possible to gain a sense of the distribution of the parameters *k*_31_, *V*_max_, and *K*_M_ within the population as for those parameters estimated from the individual timecourse data, *k*_12_ and *k*_21_, as illustrated in Figure 5.

The parameters *k*_12_, *k*_21_, *k*_31_, *V*_max_, and *K*_M_ were estimated from FCR vs. *x*_1,E_ and *T*_½_ vs. *x*_1,E_ data, simultaneously. Waldmann and Strober (1969) provide plots of FCR and *T*_½_ vs. plasma endogenous IgG concentration in g l^−1^. The data were extracted using the Digitizer tool in OriginPro^1^. The plasma IgG concentration in g l^−1^ was converted to µmol l^−1^ by dividing by the molar mass of IgG, 0.15 g µmol^−1^. The concentration was then multiplied by an average plasma volume of 3 l (Solomon et al., 1963) to obtain the quantity *x*_1,E_ in µmol.

The FCR and *T*_½_ data were fitted simultaneously by the model outputs described in Equations (11, 14). The parameter values were estimated by minimizing the sum of squared residual errors between the model outputs and the measured values, using the function NonlinearModelFit in Mathematica^2^. Due to the different scales of the parameters (0.02 < FCR < 0.17 day^−1^; 10 < *T*_½_ < 72 days) the *T*_½_ data points were assigned different weights to the FCR data points. It was assumed that the standard deviation of the residual errors is of the order of the size of the measured values, for both FCR and *T*_½_, respectively. Therefore, the weights given to the *T*_½_ data points were equal to the squared mean of the measured *T*_½_ values divided by the squared mean of the measured FCR values, and the weights given to the FCR data points were set to 1. Using this approach, the variance of the *T*_½_ residuals was assumed to be 1.08 × 10^5^ times the variance of the FCR residuals.

**Figure 7:**
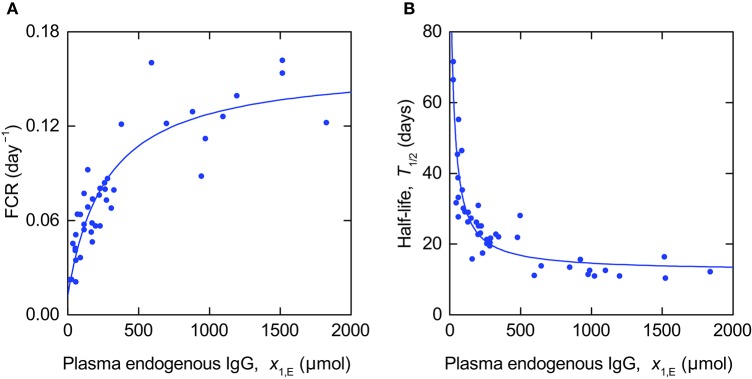
**Expressions for (A)** FCR (Equation 11) and **(B)** *T*_½_ (Equation 14) fitted to data from Waldmann and Strober (1969).

The data and model fits are shown in Figure 7. The parameter estimates and their standard errors are given in Table 4. The standard errors are almost as large as, or larger than, the estimates themselves for the parameters *k*_12_ and *k*_21_, indicating that these parameters cannot be estimated with a reasonable level of precision. This may be because the measured output *T*_½_ is insensitive to variations in the parameters *k*_12_ and *k*_21_ or due to correlations between the parameters.

**Table 4:** **Parameter estimates and standard errors estimated from FCR and ***T***½ data**.

**Parameter**	**Units**	**Estimate**	**Standard error (SE)**	**95% confidence interval**
*k*_31_	day^−1^	0.159	0.0111	(0.137, 0.181)
*V*_max_	µmol day^−1^	40.0	10.5	(19.1, 60.9)
*K*_M_	µmol	272	55.4	(162, 382)
*k*_12_	day^−1^	0.158	0.155	(−0.150, 0.467)
*k*_21_	day^−1^	0.187	0.231	(−0.273, 0.647)

#### 3.3.1. Sensitivity to model parameters and parameter correlations

**Figure 8:**
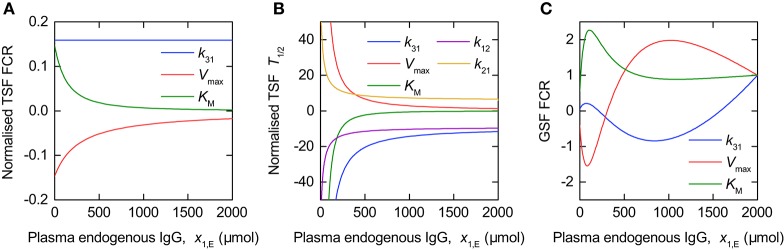
**Traditional sensitivity functions (TSFs) of (A)** FCR and **(B)** *T*_½_ and generalized sensitivity functions (GSFs) of **(C)** FCR with respect to model parameters.

The sensitivity of the outputs FCR and *T*_½_ to the model parameters is illustrated by the TSFs shown in Figure 8. In Figure 8 the TSFs are evaluated for the parameter estimates given in Table 4. Due to the wide range in parameter estimates (0.158–272), each TSF is multiplied by the value of the corresponding parameter estimate; thus the normalized TSF can be seen as representing the sensitivity of the output to variation in a parameter proportional to its value (for the particular parameter values used here). The GSFs of the FCR with respect to the parameters *k*_31_, *V*_max_, and *K*_M_ were calculated for the parameter estimates in Table 4. We assume measurements taken in intervals of 10 µmol between 0 µmol and 2000 µmol plasma endogenous IgG, *x*_1,E_, in order to get an approximately smooth function. Again we assume that the variances of the errors are equal over all concentrations, such that the variance terms cancel out in the definition of GSF (see Equations 26, 27).

Figure 8A shows the TSFs of the FCR with respect to the parameters *k*_31_, *V*_max_, and *K*_M_, evaluated for the parameter estimates in Table 4. The plot shows that, for the parameter vector used, the FCR is sensitive to all three parameters over the range of plasma endogenous IgG concentrations measured, with greater sensitivity to *K*_M_ and *V*_max_ at smaller concentrations. The similarity between the TSFs of the FCR with respect to *K*_M_ and *V*_max_, respectively, may cause high correlation between these two parameters.

Figure 8C shows the GSFs of the FCR with respect to *k*_31_, *V*_max_, and *K*_M_. The GSFs show a similar pattern to those of the timecourse observations shown in Figure 6, however the larger magnitude of oscillation indicates a strong correlation between the parameters. The GSFs indicate that, for the parameter vector used, measurements at very small quantities of plasma endogenous IgG, below around 100 µmol, have the greatest influence on the estimate of *K*_M_, then the region between around 100 and 1,000 µmol has the greatest influence over the estimate of *V*_max_, with, finally, the information on *k*_31_ available at the remaining larger quantities. This interpretation is consistent with the TSFs given in Figure 8A: the sensitivity to *K*_M_ decreases rapidly at small quantities, followed by the sensitivity to *V*_max_, with the sensitivity to *k*_31_ constant, that is insensitive to the quantity *x*_1,E_ itself.

Figure 8B shows the TSFs of *T*_½_ with respect to the parameters *k*_12_, *k*_21_, *k*_31_, *V*_max_, and *K*_M_. The plot shows that *T*_½_ is much more sensitive to all of the parameters at smaller quantities of endogenous IgG. The similarity between the trajectories of the TSFs with respect to all parameters suggests that they are highly correlated. It was not possible to calculate the GSFs of *T*_½_ due to the Fisher information matrix being ill-conditioned, such that the inverse could not be computed. If we attempt to estimate all five parameters from the *T*_½_ data alone, we obtain standard errors of the order of 10 × 106 and higher, and correlation coefficients of 1 or −1 between the parameters.

When the parameters are estimated from FCR vs. *x*_1,E_ and *T*_½_ vs. *x*_1,E_ simultaneously, we find high correlation coefficients between the parameters, with the correlation matrix given by:

$$\begin{array}{l} \begin{array}{ccccccccccccc} \; & \; & \; & k_{31} & \; & V_{\text{max}} & \; & K_{\text{M}} & \; & \; & k_{12} & \; & k_{21} \end{array}\\ \begin{array}{cc} \begin{array}{c} k_{31}\\ V_{\text{max}}\\ K_{\text{M}}\\ k_{12}\\ k_{21} \end{array} & \left(\begin{array}{ccccc} 1 & 0.928 & 0.839 & -0.290 & -0.300\\ 0.928 & 1 & 0.976 & -0.228 & -0.253\\ 0.839 & 0.976 & 1 & -0.0890 & -0.115\\ -0.290 & -0.228 & -0.0890 & 1 & 0.996\\ -0.300 & -0.253 & -0.115 & 0.996 & 1 \end{array}\right) \end{array}. \end{array}$$

Parameters *k*_31_, *V*_max_, and *K*_M_ are highly correlated pairwise, with the strongest correlation between *K*_M_ and *V*_max_. *k*_12_ and *k*_21_ are highly correlated with one another (correlation coefficient of 0.996) and not with the other parameters, explaining why they cannot be estimated with a reasonable level of precision from these data.

### 3.4. Simulations of IgG responses in IgG multiple myeloma

In order to simulate monoclonal IgG responses in IgG multiple myeloma, the model of endogenous IgG metabolism given by Equation (1) needs to explicitly account for monoclonal IgG produced by the malignant plasma cells, and polyclonal IgG produced by healthy plasma cells, since both types of IgG undergo the same processes of recycling and elimination and therefore one is influenced by the other. The dynamics of monoclonal and polyclonal IgG in an IgG myeloma patient may be described by:

(28) $$\begin{array}{l} {\dot{x}}_{1,\text{m}}(t)=-\left(k_{21}+k_{31}-\frac{V_{\text{max}}}{K_{\text{M}}+x_{1,\text{m}}(t)+x_{1,\text{p}}(t)}\right)x_{1,\text{m}}(t)\\ \;+\;k_{12}x_{2,\text{m}}(t)+I_{\text{m}}(t)\\ {\dot{x}}_{2,\text{m}}(t)=k_{21}x_{1,\text{m}}(t)-k_{12}x_{2,\text{m}}(t)\\ {\dot{x}}_{1,\text{p}}(t)=-\left(k_{21}+k_{31}-\frac{V_{\text{max}}}{K_{\text{M}}+x_{1,\text{m}}(t)+x_{1,\text{p}}(t)}\right)x_{1,\text{p}}(t)\\ \;+\;k_{12}x_{2,\text{p}}(t)+I_{\text{p}}(t)\\ {\dot{x}}_{2,\text{p}}(t)=k_{21}x_{1,\text{p}}(t)-k_{12}x_{2,\text{p}}(t), \end{array}$$

where *x*_1,m_(*t*) and *x*_2,m_(*t*) are the quantities of monoclonal IgG in plasma and in the peripheral space, respectively, *x*_1,p_(*t*) and *x*_2,p_(*t*) are the quantities of polyclonal IgG in plasma and in the extravascular space, respectively, *I*_m_(*t*) is the production rate of monoclonal IgG in µmol day^−1^, *I*_p_(*t*) is the production rate of polyclonal IgG in µmol day^−1^, and all other parameters are as previously defined.

It is assumed that the production rate of monoclonal IgG, *I*_m_(*t*), is determined by the number of myeloma cells, or tumor burden. Modeling the response of the myeloma cell population under therapy is in itself a significant problem. Here we assume a highly simplified, phenomenological model which nevertheless shows good qualitative agreement with responses seen in real patients. In the following simulations the production rate of monoclonal IgG, *I*_m_(*t*), is given by:

(29) $$I_{\text{m}}(t)=\left(I_{\text{m},0}-I_{\text{m},\infty }\right)\text{ exp }\left(-k_{\text{kill}}t\right)+I_{\text{m},\infty },$$

where *I*_m_(0) = *I*_m,0_ µmol day^−1^, *I*_m_(*t*) tends to *I*_m,∞_ µmol day^−1^ for large *t*, and *k*_kill_ day^−1^ is the rate constant of tumor kill.

**Figure 9:**
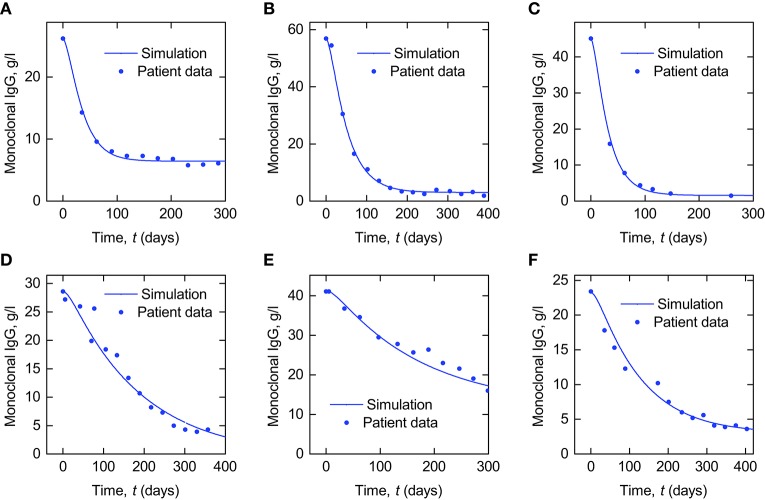
**Simulations of plasma monoclonal IgG responses in IgG myeloma alongside data from six IgG myeloma patients (A–F)**.

In Figure 9 we present simulations of monoclonal IgG responses in IgG myeloma patients during treatment. Simulations of the plasma concentration of monoclonal IgG are shown alongside plasma IgG concentrations from six IgG myeloma patients, taken from the Intergroupe Francophone du Myélome (IFM) 2009-02 clinical trial (Leleu et al., 2013). The plasma concentration of monoclonal IgG was measured by serum protein electrophoresis at regular intervals during treatment. The simulated quantities of monoclonal and polyclonal IgG are defined by Equation (28), with the monoclonal IgG production rate *I*_m_(*t*) given by Equation (29). The polyclonal IgG production rate *I*_p_(*t*) is assumed to remain constant at *I*_p,0_ = 15 µmol day^−1^, as in normal individuals (Waldmann and Strober, 1969). At time *t* = 0 the system is assumed to be in steady state, such that the initial conditions of monoclonal and polyclonal IgG are given by:

(30) $$\begin{array}{l} x_{1,\text{m}}(0)=\frac{I_{\text{m},0}}{I_{0}}\frac{\begin{array}{c} -k_{31}K_{\text{M}}+I_{0}+V_{\text{max}}\\ +\;\sqrt{4k_{31}K_{\text{M}}I_{0}+{\left(-k_{31}K_{\text{M}}+I_{0}+V_{\text{max}}\right)}^{2}} \end{array}}{2k_{31}}\\ x_{2,\text{m}}(0)=\frac{k_{21}}{k_{12}}x_{1,\text{m}}(0)\\ x_{1,\text{p}}(0)=\frac{I_{\text{p},0}}{I_{0}}\frac{\begin{array}{c} -k_{31}K_{\text{M}}+I_{0}+V_{\text{max}}\\ +\;\sqrt{4k_{31}K_{\text{M}}I_{0}+{\left(-k_{31}K_{\text{M}}+I_{0}+V_{\text{max}}\right)}^{2}} \end{array}}{2k_{31}}\\ x_{2,\text{p}}(0)=\frac{k_{21}}{k_{12}}x_{1,\text{p}}(0) \end{array}$$

where *I*_0_ = *I*_m,0_ + *I*_p,0_.

In order to convert the quantity of plasma monoclonal IgG in µmol into concentration in g l^−1^ the simulated quantity was multiplied by the molecular weight of IgG, 0.15 g µmol^−1^, and divided by an average plasma volume of 3 l. For the parameters *k*_12_ and *k*_21_ the mean values of the respective parameter estimates from the individual fits in Section 3.2 are used; for the parameters *k*_31_, *V*_max_, and *K*_M_ the values estimated from FCR and *T*_½_ vs. *x*_1,E_ in Section 3.3 are used. The parameters of the model describing the monoclonal IgG production rate, given by Equation (29), namely *I*_m,0_, *I*_m,∞_ and *k*_kill_, were manually adjusted in order to produce simulations that approximately replicate the responses seen in patients. The parameter values used are provided in Table 5.

**Table 5:** **Parameter values used to produce the simulations in Figure **9****.

**Parameter**	**Units**	**Panel**					
		**A**	**B**	**C**	**D**	**E**	**F**
*I*_m,0_	µmol day^−1^	61	152	116	68	105	53
*I*_m,∞_	µmol day^−1^	11.5	5	2.5	0	24	5
*k*_kill_	day^−1^	0.055	0.03	0.07	0.007	0.0065	0.01
*I*_p,0_	µmol day^−1^	15	15	15	15	15	15
*k*_12_	day^−1^	0.38	0.38	0.38	0.38	0.38	0.38
*k*_21_	day^−1^	0.42	0.42	0.42	0.42	0.42	0.42
*k*_31_	day^−1^	0.16	0.16	0.16	0.16	0.16	0.16
*V*_max_	µmol day^−1^	40	40	40	40	40	40
*K*_M_	µmol	270	270	270	270	270	270

The purpose of these simulations is to demonstrate how the model analyzed in this paper may be used in the future to investigate monoclonal IgG responses in IgG myeloma. Patient data are presented alongside the simulations to show that they provide good qualitative agreement with in vivo responses, supporting the suitability of the model for future investigations. In these simulations, the initial and final monoclonal IgG production rates, *I*_m,0_ and *I*_m,∞_, and the rate at which the monoclonal IgG production rate falls during treatment, *k*_kill_, have been varied, whilst the parameters of the metabolic model have been fixed. In reality there will be inter-patient variability in both the parameters of the tumor response model and the metabolic model. Here we have not explicitly modeled the response in the myeloma cell population itself—only the production rate of monoclonal IgG. Additional assumptions are required in order to investigate the relationship between the tumor response and the serum monoclonal IgG response. The simplest approach is to assume that the rate of synthesis per myeloma cell is constant and as such the total body synthesis rate *I*_m_(*t*) is directly proportional to the total myeloma cell population. It is possible that the cellular synthesis rate may vary over the course of treatment; however studies of in vitro IgG synthesis in plasma cells taken from IgG myeloma patients have shown that, whilst the cellular synthesis rate varies between patients, for an individual patient it remains constant over a period of months (Salmon and Smith, 1970). In the present study we have also assumed constant polyclonal IgG production, however it is known that normal plasma cells in the bone marrow are frequently suppressed by the clonal cell presence. If we assume that the cellular rate of IgG synthesis remains constant in normal plasma cells then suppression of these cells likely contributes to a decrease in overall polyclonal IgG synthesis. There are several complex mechanisms involved, but fundamentally the suppression of polyclonal cells is believed to be due to competition between monoclonal and polyclonal cells for survival niches in the bone marrow microenvironment (Paiva et al., 2011). Mathematically modeling normal plasma cell suppression could be the subject of future research. It has also been assumed that the system is in steady state at the commencement of treatment. Future studies will be required to validate this assumption or employ alternative models in which the tumor is growing initially, and to assess the impact of the chosen assumptions on any conclusions drawn from the simulations.

## 4. Discussion

The aim of this study was to analyse a previously published model of endogenous IgG metabolism and available measurements in humans with respect to parameter identifiability. The model was linearized to replicate experimental conditions in which small doses of administered IgG exhibit linear timecourse responses. The linearized model was then analyzed in terms of parameter structural identifiability and parameter sensitivity. The analyses show that certain important parameters are not structurally identifiable from an individual's timecourse response; however they are structurally identifiable using the relationships between the FCR and *T*_½_, respectively, and the quantity of endogenous IgG in plasma, which is assumed to remain in steady state.

A limitation of the linear model of an individual's timecourse response, given by Equations (8–10), is that the parameters *k*_31_, *V*_max_, and *K*_M_ are structurally unidentifiable. It is not known whether these parameters are structurally identifiable in the nonlinear model of coupled radiolabeled and endogenous IgG responses given by Equations (5–7). There are two reasons this approach was not pursued here: firstly, structural identifiability analysis of a four-state nonlinear model with rational terms presents a more challenging problem; secondly, if the nonlinear model were found to be structurally identifiable, the responses available nonetheless do not demonstrate nonlinear behavior due to the small doses of radiolabeled IgG administered (see Figure 3)—therefore the parameters representing nonlinear behavior may not be practically identifiable by fitting the nonlinear model, and there is an increased risk of fitting the noise in the data with the increased model complexity.

Structural identifiability analysis alone does not imply that parameters can necessarily be estimated in practice. In this paper two sensitivity functions were investigated: the traditional sensitivity function (TSF) and generalized sensitivity function (GSF). TSFs and GSFs were computed and plotted for the timecourse outputs *y*_1_(*t*) and *y*_2_(*t*), using the parameter values estimated from the timecourse data of seven individuals. The TSFs show that the measured timecourse outputs are sensitive to the model parameters over the duration of the experiment, which is different for each individual. In addition, the GSFs show the influence of the duration of observation taking into account correlation between the parameters; for example, subject A is observed over a relatively short time period compared to subjects B and C, considering its slower dynamics. The GSF curves of *y*_1_(*t*) for subject A show a larger magnitude of oscillation than those of subjects B and C; however when the duration of observation is increased for subject A, the GSFs of *y*_1_(*t*) are remarkably similar for the three subjects. This would suggest that the estimation of the parameters for subject A would benefit from a longer duration of observation. If the tracer experiments were to be repeated, the insights obtained from the GSFs in particular could be used to inform the sampling grid of measurements. With the data that are currently available, *k*_12_, *k*_21_, and the FCR are estimated with a good level of precision for all subjects.

TSFs and GSFs were also calculated for the relationship between the FCR and *x*_1,E_. The TSFs indicate that the FCR is most sensitive to the parameters *V*_max_ and *K*_M_ at small concentrations of plasma endogenous IgG; this is also indicated in the GSFs, which show that the information on *K*_M_ is concentrated very close to zero, followed by *V*_max_ and finally *k*_31_. The TSFs for the *T*_½_ imply high correlation between all the parameters. From the simultaneous estimation of the parameters from both FCR and *T*_½_ data, the parameters *k*_31_, *V*_max_, and *K*_M_ are estimated with a reasonable level of precision. We notice that the estimated values of *k*_12_ and *k*_21_ are quite different from the averages of the estimates of the same parameters obtained from fitting individual timecourses. From the FCR and *T*_½_ data, the 95% confidence interval estimates of *k*_12_ and *k*_21_ are given by (−0.150, 0.467) and (−0.273, 0.647), respectively. These large intervals containing zero imply that the parameters *k*_12_ and *k*_21_ are not well estimated from these data. The estimates of *k*_12_ and *k*_21_ from the FCR and *T*_½_ data are very highly correlated with one another, but not with the remaining three parameters; this offers an explanation for why they are not well estimated from these data. Nevertheless, the 95% confidence intervals for *k*_12_ and *k*_21_ contain the averages of the individual estimates from timecourse data, 0.38 and 0.42, respectively. If we fix *k*_12_ to 0.38 and *k*_21_ to 0.42 in the estimation from the FCR and *T*_½_ data, the newly obtained parameter estimates of *k*_31_, *V*_max_, and *K*_M_ are 0.161, 45.6, and 307, respectively. These values fall well within the confidence intervals of the previous estimation where *k*_12_ and *k*_21_ are unconstrained.

Table 6 compares previously published parameter values alongside the parameter values estimated in this paper. For the parameters *k*_12_ and *k*_21_ the mean value of the parameter among the seven subjects is chosen to represent the average; for the parameters *k*_31_, *V*_max_, and *K*_M_ the values estimated from FCR and *T*_½_ vs. *x*_1,E_ data from many subjects are taken to represent the population average. At first glance the newly estimated parameter values are not wildly dissimilar to the previously published values. Waldmann and Strober (1969) give the values of *k*_31_ = 0.18 day^−1^ and *V*_max_/*w* = 147 mg day^−1^ kg^−1^, where *w* is body weight in kg. Assuming a 70 kg human, this is equivalent to *V*_max_ = 68.6 µmol day^−1^. The value of *k*_31_ was obtained ‘from the asymptotic value of the plot of the IgG fractional catabolic rate versus its concentration’. These are the same data that were utilized in this paper as described in Section 2.1. The authors subtracted the value of the FCR for each individual from 0.18 to find the fractional recycling rate. The fractional recycling rate (FRR) is thus given by:

(31) $$\text{FRR}=k_{31}-\text{FCR}=k_{31}-\left(k_{31}-\frac{V_{\text{max}}}{K_{\text{M}}+x_{1,\text{E}}}\right)=\frac{V_{\text{max}}}{K_{\text{M}}+x_{1,\text{E}}}.$$

**Table 6:** **Comparison with published parameter values**.

**Parameter**	**Units**	**Previously published values**			**This paper**
		**Waldmann and Strober (1969)**	**Kim et al. (2007)**	**Hattersley et al. (2013)**	
*k*_12_	day^−1^	–	0.158	0.41	0.38
*k*_21_	day^−1^	–	0.156	0.51	0.42
*k*_31_	day^−1^	0.18	0.18^†^	0.13	0.16
*V*_max_	µmol day^−1^	68.6^*^	68.6^*^^†^	103	40
*K*_M_	µmol	–	420^**^	530	270

* *Assuming 70 kg human*.

** *Assuming 3 l plasma volume*.

† *Taken from Waldmann and Strober (1969)*.

They then multiplied the plasma concentration of endogenous IgG by the plasma volume per kg of body weight for each individual to get the quantity of endogenous IgG in plasma per kg of body weight, *x*_1,E_/*w*. Finally the authors multiplied the FRR by *x*_1,E_/*w* to obtain the absolute recycling rate per kg of body weight, which we will call ARR. They then plotted the reciprocal of the ARR against the reciprocal of *x*_1,E_/*w* to obtain a straight line relationship, given by:

(32) $$\frac{1}{\text{ARR}}=\frac{K_{\text{M}}}{V_{\text{max}}}\frac{w}{x_{1,\text{E}}}+\frac{w}{V_{\text{max}}}.$$

From the intercept the authors obtained the value of *V*_max_/*w* = 147 mg kg^−1^ day^−1^.

Kim et al. (2007) provide values for all model parameters. Again using the FCR vs. endogenous IgG concentration data first published by Waldmann and Strober (1969) and described here in Section 2.1, Kim et al. (2007) estimate *K*_M_/*w* using a least-squares fitting. The equation fitted to the data is

(33) $$\text{FCR}=k_{31}-\frac{V_{\text{max}}/w}{\frac{v_{1}}{w}\left(\frac{K_{\text{M}}}{v_{1}}+\frac{x_{1,\text{E}}}{v_{1}}\right)},$$

where *v*_1_ is the plasma volume. The authors fit Equation (33) to FCR vs. *x*_1,E_/*v*_1_ to obtain *K*_M_/*v*_1_ whilst fixing the remaining parameters as follows: *k*_31_ = 0.18 day^−1^ (Waldmann and Strober, 1969), *V*_max_/*w* = 0.98 µmol kg^−1^ (Waldmann and Strober, 1969), and *v*_1_/*w* = 0.042 l kg^−1^ (Waldmann and Terry, 1990). By this approach the authors obtain a value of *K*_M_/*v*_1_ = 140 µmol l^−1^. Assuming a plasma volume of 3 l this is equates to *K*_M_ = 420 µmol. The authors also estimate the parameters *k*_12_ and *k*_21_ by curve fitting to tracer experiment data from Waldmann and Terry (1990).

The parameter values provided by Hattersley et al. (2013) were obtained by methods described in Hattersley (2009). Hattersley (2009) estimates parameters *k*_12_ and *k*_21_ by fitting the model to tracer experiment data in Waldmann and Strober (1969). For the remaining model parameters, *k*_31_, *V*_max_, and *K*_M_, the author takes a completely different approach, fitting the model directly to serum IgG data from an IgG myeloma patient, assuming a delayed logistic function to describe the production of tumor-produced IgG. For this approach, the parameters *k*_12_, *k*_21_, and *v*_1_ were fixed.

In this paper data from a number of subjects have been used for parameter estimation: parameters *k*_12_ and *k*_21_ were estimated individually for seven subjects and parameters *k*_31_, *V*_max_, and *K*_M_ were estimated from the pooled data of around 40 subjects. In order to make predictions of IgG responses in IgG multiple myeloma that can be generalized across patients, a model which characterizes average, or expected, behavior is advantageous. One of the limitations of this study is that a full population approach has not been taken. Fitting the timecourse data individually and summarizing the parameter estimates by the sample mean or median can be seen as a two-stage approach, and fitting the FCR and *T*_½_ data as though they arose from an individual is essentially a naive pooled approach; these approaches have been shown to be inferior to a full population approach (Wright, 1998). A population approach has not been taken here due to the limited data that are available in the literature. In future studies it may be possible with further experiments to apply a full population approach and gain information on the distribution of all parameters within the population. Furthermore, the work presented here could be enhanced with a simulation study in which parameters are estimated from synthetic data, in order to provide additional understanding of the identification problem and inform the design of future experiments.

In Section 3.4 we have shown how the model can be extended to simulate monoclonal IgG responses in IgG myeloma. The assumptions behind these simulations are discussed in detail in that section. In IgG myeloma patients the serum monoclonal IgG response is used as a surrogate for the tumor response to treatment; however the relationship between the tumor response and the monoclonal IgG response is inevitably influenced by the natural elimination of IgG from the body, which is known to have a non-constant relationship with serum IgG concentration. It is our intention that the model analyzed in this paper can be used in the future as the basis of more detailed investigations into the dynamics of IgG responses in IgG multiple myeloma; for example, is the prediction of long-term outcomes by monoclonal IgG responses influenced by the concentration-dependent and comparatively long half-life of the protein? Such future studies could impact upon how responses to treatment are assessed in patients.

In addition, the concentration-dependent elimination of IgG may be implicated in the pharmacokinetics of monoclonal antibody (mAb) agents for multiple myeloma, for example daratumumab, which is currently undergoing evaluation in patients with relapsed or refractory disease (Costello, 2017). Due to FcRn-mediated recycling, the longevity of daratumumab is predicted to be shorter in patients with high monoclonal IgG loads whereas low monoclonal IgG concentrations may favorably affect the pharmacokinetic profile of the agent, such that doses could be administered at less frequent intervals. The pharmacokinetics of various mAbs have been well studied; however the use of mAbs in multiple myeloma is recent and the dynamic response of the tumor-produced IgG in IgG myeloma patients adds an additional level of complexity. With the appropriate data it would be highly interesting from a pharmacological point of view to couple mathematical models of the mAb disposition and the tumor-produced IgG response, which is in turn directly affected by the efficacy of the agent.

IgG metabolism is implicated in other medical applications beyond patient monitoring in multiple myeloma, including antibody mediated rejection of transplants, infection and intravenous IgG (IVIG) therapy. In medical applications, mathematical models can be used to investigate biomedical systems *in silico*, allowing many scenarios and interventions to be simulated whilst avoiding the costs associated with human and animal experimentation. For biomedical applications, the parameter values used are of high importance as they can greatly affect conclusions drawn from simulations. In this paper structural identifiability analysis and sensitivity analysis are presented as a first stage in the model validation process, showing which model parameters are identifiable from which measured outputs. Together structural identifiability analysis and sensitivity analysis can be used to inform parameter estimation and improve confidence in the methodology used. In order for the model to applied in other biomedical scenarios, validation against patient data would be necessary. A limitation of the model analyzed here is that it may not contain a sufficient level of detail for all applications. Future work may involve comparing this simple model with more complex models that are based closely on the biological mechanisms, such as the model presented by Ferl et al. (2005).

## 5. Conclusions

In this research a previously published model of endogenous IgG metabolism in humans has been analyzed and parameter values estimated using limited data from the literature. The analyses herein provide an understanding of how parameter estimates have been obtained and sources of uncertainty; for future applications in which the parameter values themselves are of key importance, an understanding of how they were obtained and why is crucial. The parameterized model can have a wide-ranging impact in studies of endogenous IgG metabolism in biomedical applications, not limited to investigations of IgG as a response marker in IgG multiple myeloma, supporting therapeutic interventions and patient monitoring.

## Author contributions

FK performed model analyses and parameter estimation. FK, MC, and NE wrote the manuscript. MC, NE, and SHarding initiated, coordinated, and supervised the work. XL provided discussion on the clinical application of the work. XL, BA, HA, OD, TD, GF, SG, SHarel, BH, VJ, PM, VR, SS, and CT provided data from the Intergroupe Francophone du Myélome (IFM) 2009-02 clinical trial. All authors reviewed and approved the final manuscript.

## Funding

This research was supported by a Biotechnology and Biological Sciences Research Council (BBSRC) studentship, through the Midlands Integrative Biosciences Training Partnership (MIBTP), and an Engineering and Physical Sciences Research Council (EPSRC) Impact Acceleration Account (IAA) award.

### Conflict of interest statement

The authors declare that the research was conducted in the absence of any commercial or financial relationships that could be construed as a potential conflict of interest.

## Footnotes

^1^Originpro (2016). Windows. Northampton: OriginLab Corporation.

^2^Mathematica 10.4 (2016). Windows. Champaign: Wolfram Research Inc.
