# Supplementary material for: Analysis of a Compartmental Model of Endogenous Immunoglobulin G Metabolism with Application to Multiple Myeloma
Source: Front Physiol. 2017 Mar 17;8:149. doi: 10.3389/fphys.2017.00149 (PMC5355465; doi:10.3389/fphys.2017.00149)
Supplement: Supplementary file 1 [file DataSheet1.pdf]

Supplementary Material: Analysis of a Compartmental Model of Endogenous Immunoglobulin G Metabolism with Application to Multiple Myeloma

Felicity Kendrick, Neil D. Evans, Bertrand Arnulf, Hervé Avet-Loiseau, Olivier Decaux, Thomas Dejoie, Guillemette Fouquet, Stéphanie Guidez, Stéphanie Harel, Benjamin Hebraud, Vincent Javaugue, Valentine Richez, Susanna Schraen, Cyrille Touzeau, Philippe Moreau, Xavier Leleu, Stephen J. Harding and Michael J. Chappell*

*Correspondence:
Author Name: Michael J. Chappell
m.j.chappell@warwick.ac.uk

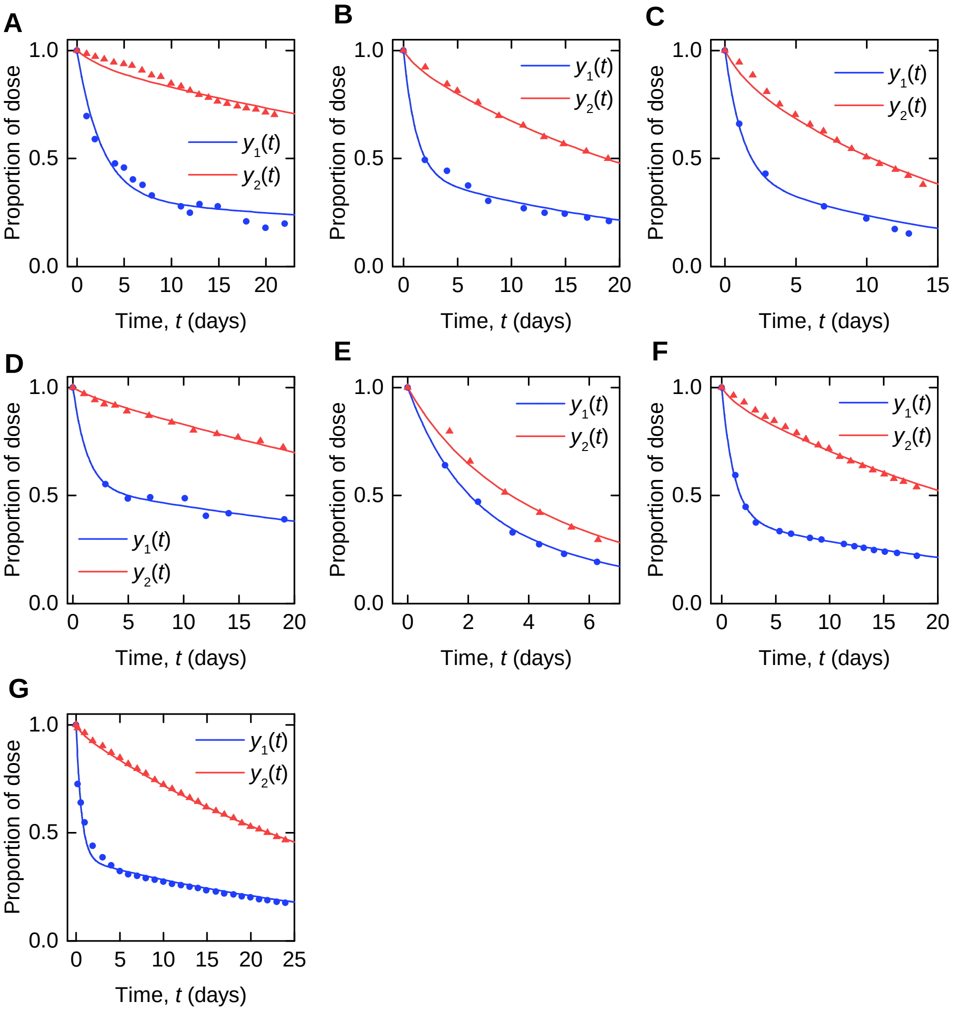

Figure S1. Timecourse fits: model fitted to timecourse data for (A–G) subjects A, B, C, D, E, F and G.

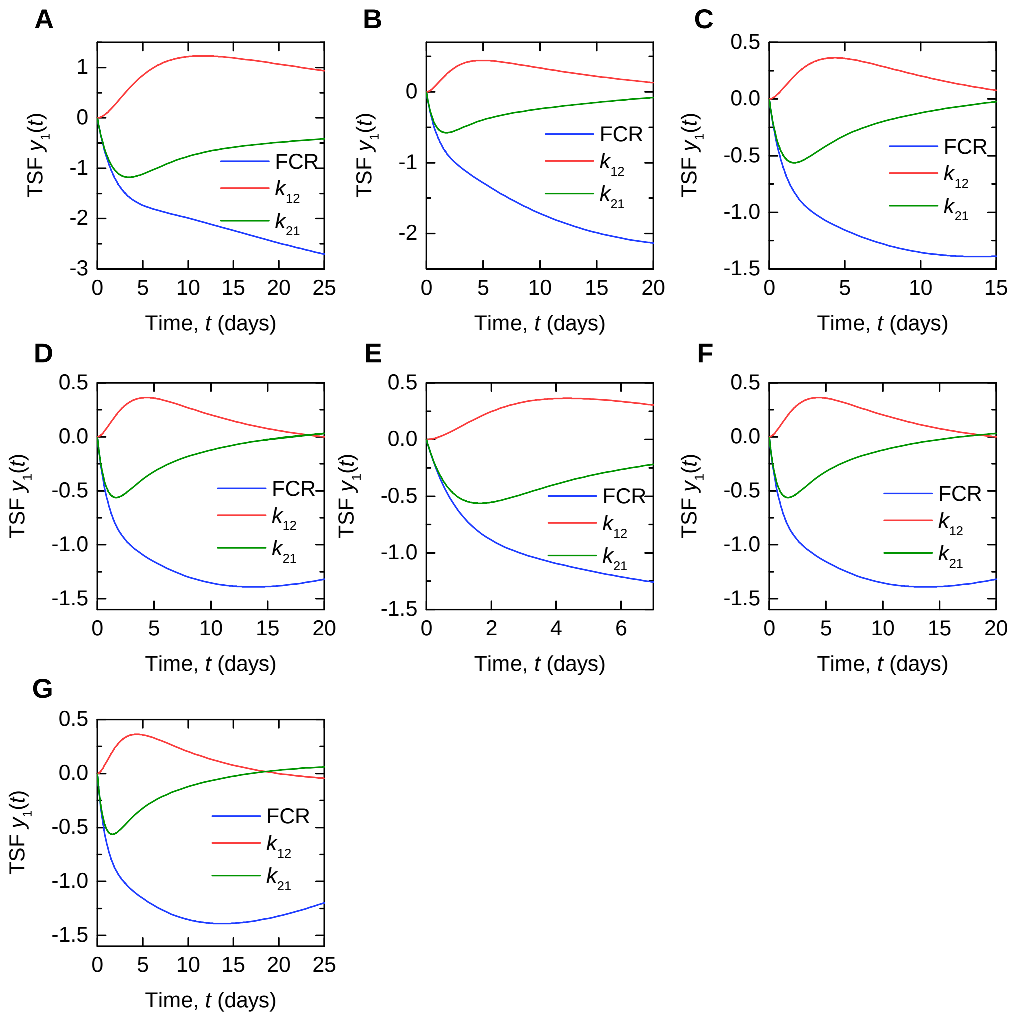

Figure S2. Traditional sensitivity functions (TSFs) of timecourse output $y_1(t)$, for (A–G) subjects A, B, C, D, E, F and G.

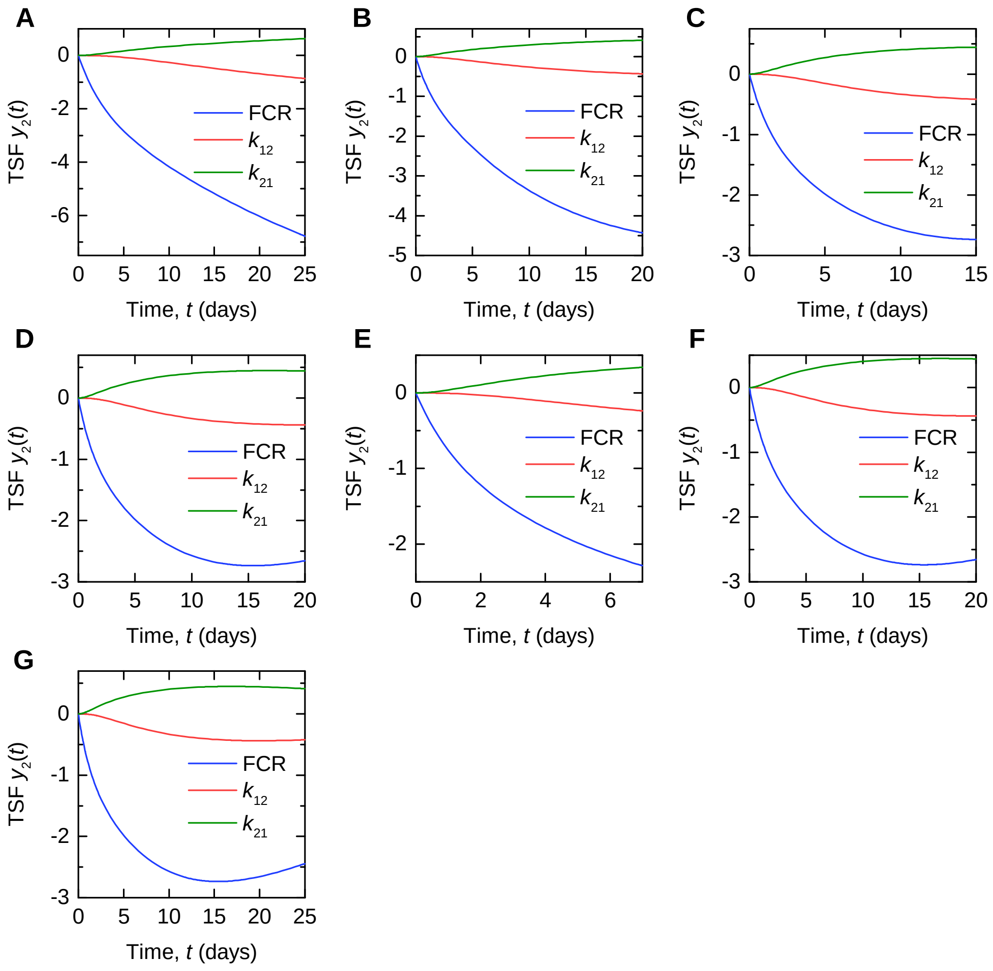

Figure S3. Traditional sensitivity functions (TSFs) of timecourse output $y_2(t)$, for (A–G) subjects A, B, C, D, E, F and G.

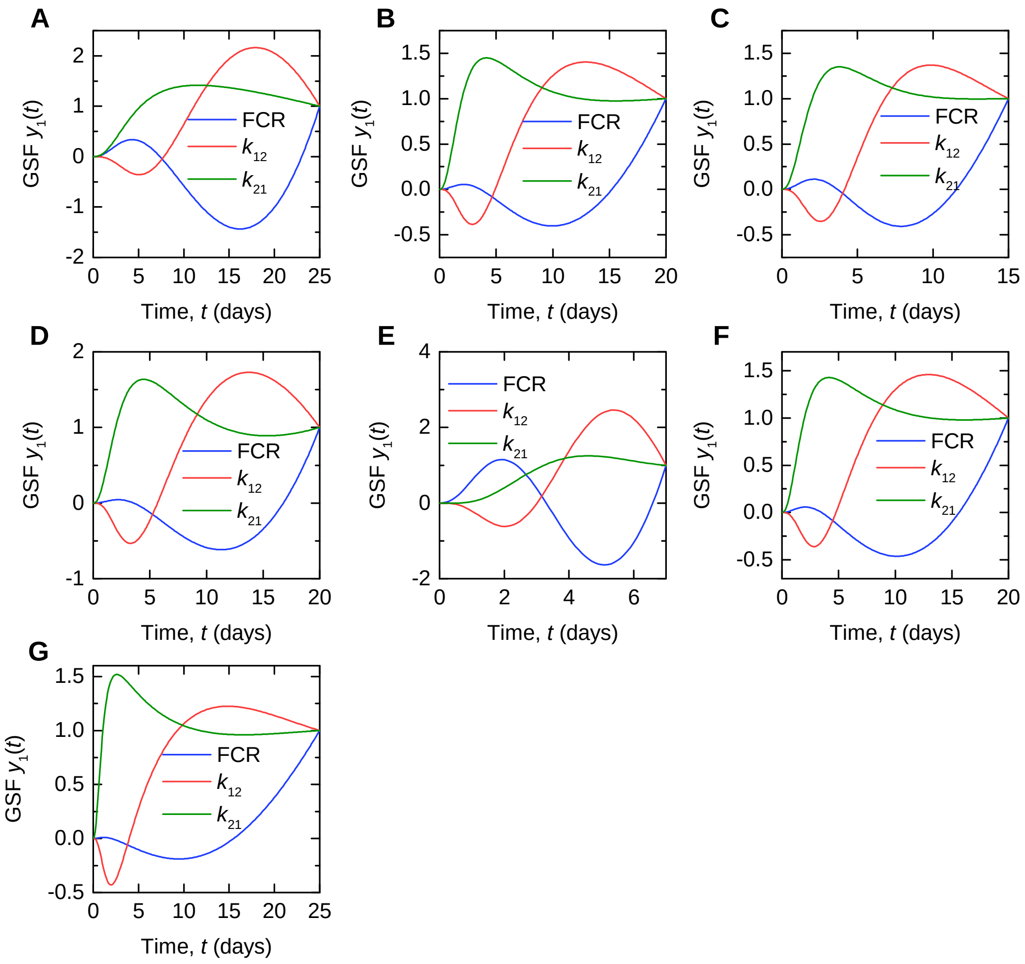

Figure S4. Generalised sensitivity functions (GSFs) of timecourse output $y_1(t)$, for (A–G) subjects A, B, C, D, E, F and G.

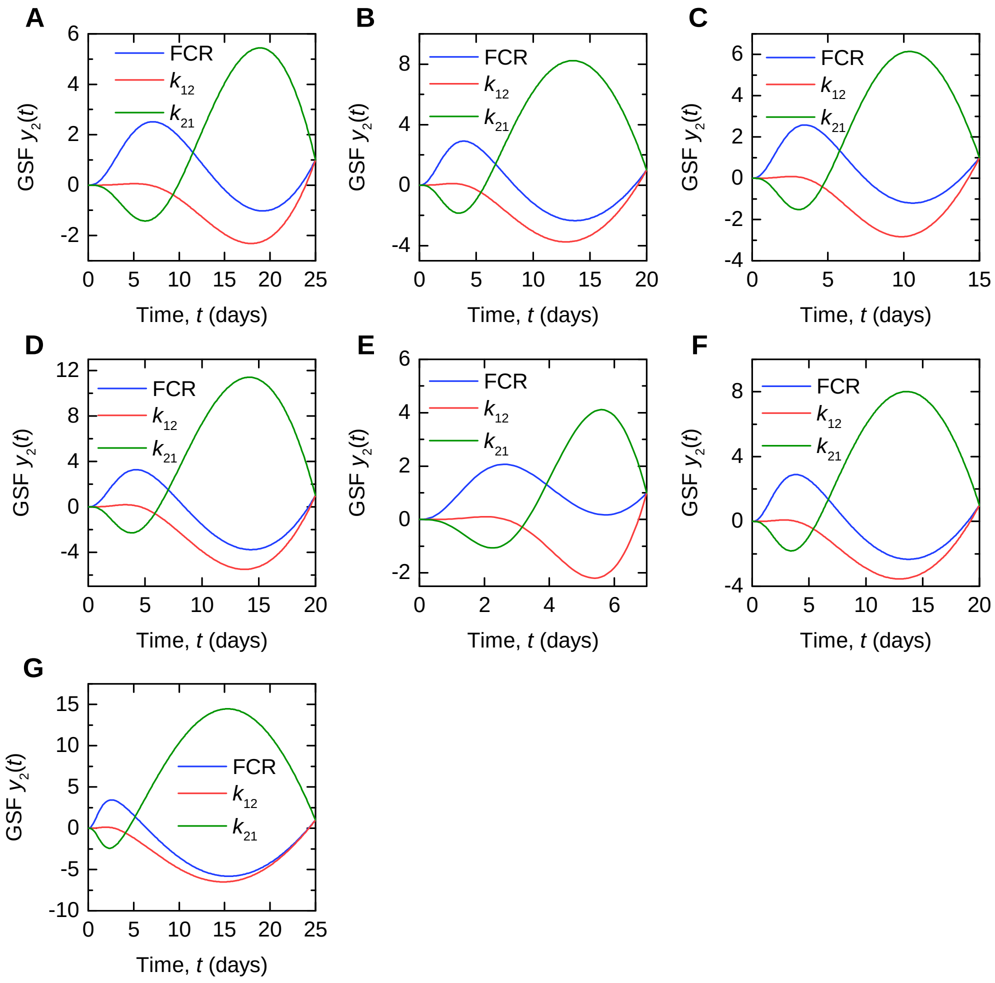

Figure S5. Generalised sensitivity functions (GSFs) of timecourse output $y_2(t)$, for (A–G) subjects A, B, C, D, E, F and G.
